# Population confidence in the health system in 15 countries: results from the first round of the People's Voice Survey

**DOI:** 10.1016/S2214-109X(23)00499-0

**Published:** 2023-12-11

**Authors:** Margaret E Kruk, Neena R Kapoor, Todd P Lewis, Catherine Arsenault, Eleni C Boutsikari, João Breda, Susanne Carai, Kevin Croke, Rashmi Dayalu, Günther Fink, Patricia J Garcia, Munir Kassa, Sailesh Mohan, Mosa Moshabela, Jacinta Nzinga, Juhwan Oh, Emelda A Okiro, Dorairaj Prabhakaran, Gillian K SteelFisher, Rosanna Tarricone, Ezequiel Garcia-Elorrio

**Affiliations:** a Department of Global Health and Population, Harvard T H Chan School of Public Health, Boston, MA, USA; b Department of Health Policy and Management, Harvard T H Chan School of Public Health, Boston, MA, USA; c Department of Global Health, George Washington University Milken Institute School of Public Health, Washington, DC, USA; d Division of Country Health Policies and Systems, WHO Athens Quality of Care Office, WHO Regional Office for Europe, Athens, Greece; e Department of Epidemiology and Public Health, Swiss Tropical and Public Health Institute and University of Basel, Basel, Switzerland; f School of Public Health, Universidad Peruana Cayetano Heredia, Lima, Peru; g Minister's Office, Ministry of Health, Addis Ababa, Ethiopia; h Public Health Foundation of India, New Delhi, India; i College of Health Sciences, University of KwaZulu–Natal, Durban, South Africa; j Health Economics Research Unit, KEMRI–Wellcome Trust Research Programme, Nairobi, Kenya; k Population and Health Impact Surveillance Group, KEMRI–Wellcome Trust Research Programme, Nairobi, Kenya; l Seoul National University College of Medicine, Seoul, South Korea; m Centre for Tropical Medicine and Global Health, Nuffield Department of Medicine, University of Oxford, Oxford, UK; n Department of Social and Political Science, Bocconi University, Milan, Italy; o Health Care Quality and Patient Safety, Institute for Clinical Effectiveness and Health Policy, Buenos Aires, Argentina

## Abstract

Population confidence is essential to a well functioning health system. Using data from the People's Voice Survey—a novel population survey conducted in 15 low-income, middle-income, and high-income countries—we report health system confidence among the general population and analyse its associated factors. Across the 15 countries, fewer than half of respondents were health secure and reported being somewhat or very confident that they could get and afford good-quality care if very sick. Only a quarter of respondents endorsed their current health system, deeming it to work well with no need for major reform. The lowest support was in Peru, the UK, and Greece—countries experiencing substantial health system challenges. Wealthy, more educated, young, and female respondents were less likely to endorse the health system in many countries, portending future challenges for maintaining social solidarity for publicly financed health systems. In pooled analyses, the perceived quality of the public health system and government responsiveness to public input were strongly associated with all confidence measures. These results provide a post-COVID-19 pandemic baseline of public confidence in the health system. The survey should be repeated regularly to inform policy and improve health system accountability.

This is the first in a **Series** of six papers about the People's Voice Survey on health system performance. All papers in the Series are available at www.thelancet.com/series/peoples-voice-survey

## Background

Confidence is the belief that one can rely on someone or something.^1^ The *Lancet Global Health* Commission on high quality health systems identified confidence as one of the outcomes of a high-quality system, along with better health and economic benefit.^2^ Public confidence in the system facilitates uptake of needed care and adherence to treatment.^3^, ^4^, ^5^, ^6^, ^7^, ^8^, ^9^, ^10^, ^11^ Trust in health systems, as in other social institutions, promotes cohesion and social order—a key rationale for universal health coverage.^12^, ^13^, ^14^ Measuring confidence promotes accountability to the population, since populations are the ultimate payers of health systems.^2^, ^15^, ^16^, ^17^, ^18^, ^19^, ^20^, ^21^ Confidence can serve as an outcome measure for policy reforms.^22^, ^23^ However, although most health systems claim to be people-centred, few measure confidence systematically or over time.

Confidence or trust in the health system has been variously defined.^24^ In this Series paper we focus on two key dimensions: health security and endorsement. Individuals' health security is defined as safety from chronic and sudden health threats through accessible, affordable, good-quality health care.^25^, ^26^ This health safety net—a guarantee of care without financial hardship—is core to the concept of universal health coverage.^21^, ^27^, ^28^ Although satisfaction has often been used as a measure of confidence, here we use endorsement of the health system, as this assessment is concrete, policy informative, and less dependent on factors outside the health system.^29^, ^30^ We examine two types of endorsement: support for the current system and its trajectory (through an assessment of performance over the past 2 years).

A key determinant of confidence is the quality of care people experience in the health system, and quality is itself a measure of system performance in people-centred health systems.^12^ Ratings of health system quality (among users and non-users) reflect an individual's own experience and experiences of their friends and family, and broader perceptions shaped by media, advertising, and the presence of alternatives. Confidence is additionally shaped by different expectations, values, and personal health status and goals.^29^ Finally, the social and political context in which health systems operate can affect population confidence.^4^, ^20^, ^22^

**Key messages**

• On average fewer than half of surveyed individuals across 15 countries were confident that they could get and afford good-quality care if sick.
• Only a quarter of respondents across countries said their system worked well. The lowest endorsement was in countries with major recent health-care challenges: Greece, the UK, and Peru.
• Countries less affected by COVID-19 and low-income countries had greater support for the notion that the health system was improving in the past 2 years.
• Wealthier respondents were more confident they could get and afford needed care. Women and people with post-secondary education, and, in some countries, young people, were less likely to endorse the health system than respondents with lower education levels, men, and people older than 30 years.
• In countries with substantial private sectors, private health systems received higher ratings than public health systems.
• Primary care services for women and children were rated more highly than services for chronic and mental health conditions in most countries.
• In combined regression analysis, health security and endorsement were strongly associated with quality of the public health system and government responsiveness to public opinion.

Most research on confidence in the health system has been restricted to high-income or middle-income countries, and key constructs have been variously defined across instruments.^31^, ^32^, ^33^ Here we present findings from a novel instrument, the People's Voice Survey (PVS)—a population-representative survey to assess health system performance from a population perspective. The survey was designed to measure core health system performance domains, such as confidence and quality, across a broad range of countries. Countries can use the PVS to track their own performance over time and benchmark their results to those of their peers. Fielding across a diverse range of countries allows closer study of how public sentiment is influenced by national context and individual factors.

Extant health systems research has measured interpersonal trust and trust in government, science, and vaccines, but less work has focused on confidence in the health system.^11^, ^34^ In this Series paper we describe people's confidence in the health system in 15 countries that conducted the first round of the PVS, representing a broad range of income levels and geographies. We also describe several measures of health system quality, which are potential drivers of confidence, including quality of the health system and primary care, government responsiveness to public input, and COVID-19 management. We explore the association between confidence and key demographic factors, and analyse the association of confidence with health system quality and responsiveness across countries. We conclude with policy and research implications.

## Survey instrument

This research was done by the Quality Evidence for Health System Transformation (QuEST) Network, a global research consortium on high-quality health systems. The PVS builds on previous literature and frameworks^17^, ^18^, ^19^, ^35^, ^36^, ^37^, ^38^ on health system quality deficits, includes new theory-grounded domains, can be used across countries at all income levels and across health system types, and is designed to be rapid and affordable to permit repeated use. Survey items in the PVS include demographics and health, use of care and system competence, care experience, and health system quality and confidence. The Harvard University Institutional Review Board deemed this research exempt from full review, and additional local ethical approval was obtained as required in implementing countries.

Detailed information on the concept, development, and fielding of the PVS is available in appendix 1 (pp 1–8). In brief, the questionnaire was developed through the use of international best practices for survey research. The content was guided by the conceptual framework of the *Lancet Global Health* Commission on high quality health systems, with question wording, response options, and sequencing informed by reviews of previous surveys used in high-income and low-income countries and by input from the PVS global development group, consisting of health system academics, managers, policy makers, and health-care users. Content validity was further tested through external peer review by health system experts in international organisations and survey methods specialists. The questionnaire was assessed for comprehension in most countries via cognitive interviews that included open-ended questions concerning key concepts. The instrument was translated into local languages by professional translators, and pretests were conducted in all settings to refine question wording and local response options. The survey was piloted in each country by the study contractor and corrections were made by local research teams before data collection began.

Data for this paper were obtained from cross-sectional surveys of a population-representative sample of adults aged 18 years and over in Argentina (Province of Mendoza), Colombia, Ethiopia, Greece, Italy, India, Kenya, Laos, Mexico, Peru, South Africa, South Korea, the USA, Uruguay, and the UK by research teams collaborating with the QuEST Network. In most countries, random probability sampling was obtained through a random digit dialling approach from mobile phone frames or overlapping mobile and fixed phone frames, if fixed phone lines were commonly used in the country. In Ethiopia, a known-list sampling approach was used from a database provided by the Ethiopian Central Statistics Agency. In Ethiopia and Kenya, where mobile telephone ownership is less than 80%, additional face-to-face household-based surveys were done in areas with lower phone use through a multistage clustered sample design to achieve national representation. In South Korea, the UK, and the USA, the survey was administered through online probability panels. Additional information on these sampling methods is available in appendix 2 (pp 9–10).

Final country samples ranged from 1001 to 2779 participants. Most surveys were administered by survey research firms Ipsos or SSRS between May 9, 2022 and July 16, 2023. Post-stratification weights were constructed on the basis of country-specific demographic variables to account for differences in sample design and probability of selection (appendix 2 pp 1–2).

## Measures

Confidence in the health system includes having health security and being supportive of the system as it currently functions. The first of these measures, health security, is the judgement that the health system can respond to one's health needs and that one can afford the needed care. To this end, we calculate the percentage of respondents answering “somewhat” or “very confident” to the questions “how confident are you that you would receive good quality health care if you became very sick?” and “[could you] afford the health care you needed if you became very sick?”. We also calculate the percentage of people agreeing with both statements; this combination can function as a people-reported measure of universal health coverage.^19^

The second way we measure confidence is through endorsement of the direction and current functioning of the health system. For the first endorsement, we calculated the percentage of respondents agreeing that the health system improved in the past 2 years (*vs* staying the same or worsening). This measure reflects the trajectory of the health system and will therefore be sensitive to reforms and health shocks, such as the COVID-19 pandemic between 2020 and 2022 (this timeframe overlaps with the first wave of the PVS). The second endorsement item was agreement that the system works well as is and needs only minor reforms (*vs* major changes needed or a need to completely rebuild). This metric has been used in past health system surveys as an indicator of support for the current health system model.^37^, ^38^

We theorised that the quality of the health system and health-related governance were important health system determinants of confidence. We therefore describe population assessments of perceived quality of care: the quality of key public primary care services (with separate ratings for maternity, paediatric, chronic disease, and mental health care), and the quality of public, private, or other health-care systems (eg, social security systems). The public system described in each country varied (see appendix 1 p 2). In countries with additional major systems, such as social security systems, respondents were asked to rate those systems (appendix 1 p 2). Responses were dichotomised into “very good” and “excellent” versus “good”, “fair”, or “poor”.

**Figure 1:**
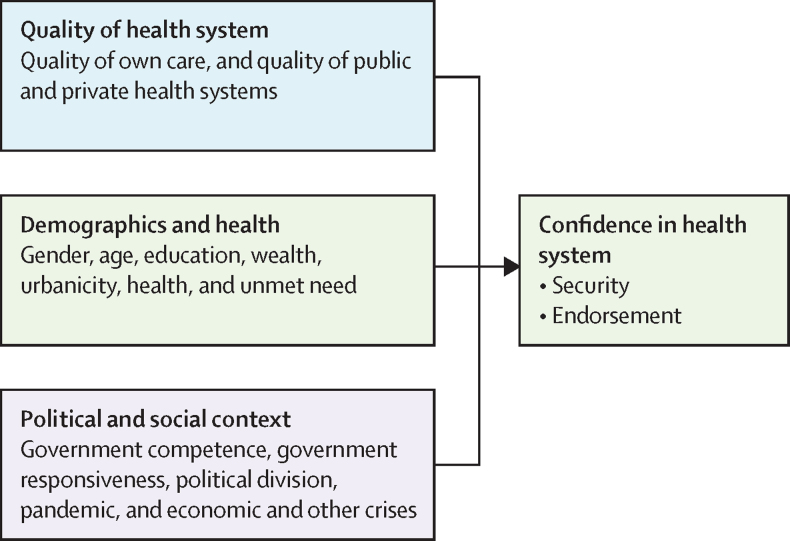
Factors influencing confidence in the health system

Demographic, social, and political factors also influence confidence (figure 1). We collected data on demographic factors such as age, self-reported gender, urbanicity, education, self-rated health, unmet need for care, and presence of chronic disease. Social and political contextual factors included whether governments sought public input for health system improvements and how well governments managed COVID-19. The latter factor would be expected to affect health system perceptions given that this round of surveys was completed between 2022 and 2023.

## Analysis

Descriptive analyses were conducted for the quality and confidence measures in each country by use of sampling and post-stratification weights (as applicable). To understand how demographic factors affected quality ratings and confidence in each country, we did separate logistic regressions. We regressed four quality and confidence outcomes in each country on five demographic factors of interest: respondent income (highest third *vs* lowest two-thirds), education (post-secondary or higher education *vs* lower education levels), urbanicity (urban *vs* rural living), age (respondents younger *vs* older than 30 years), and gender (female *vs* male), controlling for self-rated health. We dichotomised income and education at the higher levels to explore how wealthier and more educated respondents—whose contributions are essential to maintain financing for universal health coverage—rate the health system compared with individuals with lower income and education levels. We dichotomised age at 30 years to assess how young people perceive health systems, as these respondents will be future users and contributors.

We then conducted a pooled, multicountry regression analysis to examine the relationship between confidence and health system quality and responsiveness, using the explanatory framework shown in figure 1, which is based on past research and theory.^13^, ^20^, ^39^, ^40^ The key predictors of interest were ratings of the quality of public and private health systems, governments' responsiveness to public opinion, and COVID-19 management as a measure of competence. In the interest of model parsimony, quality ratings of key primary care services were not included in the model as these ratings overlapped with public health system quality. We controlled for demographic factors known to be associated with health system satisfaction or trust, such as age, education, wealth, health status (self-rated health and having a chronic disease), and unmet health-care needs in the past year.^22^, ^41^ Country fixed effects capture national economic, social, and political factors, such as government competence, government responsiveness, political crises, recessions, system reforms, health crises, and media portrayal, which have been shown to affect confidence (figure 1).^4^, ^20^, ^22^ Descriptive data for all the variables are in appendix 2 (pp 4–7). Stata version 17 was used for all analyses.

## Findings on confidence and quality

**Figure 2:**
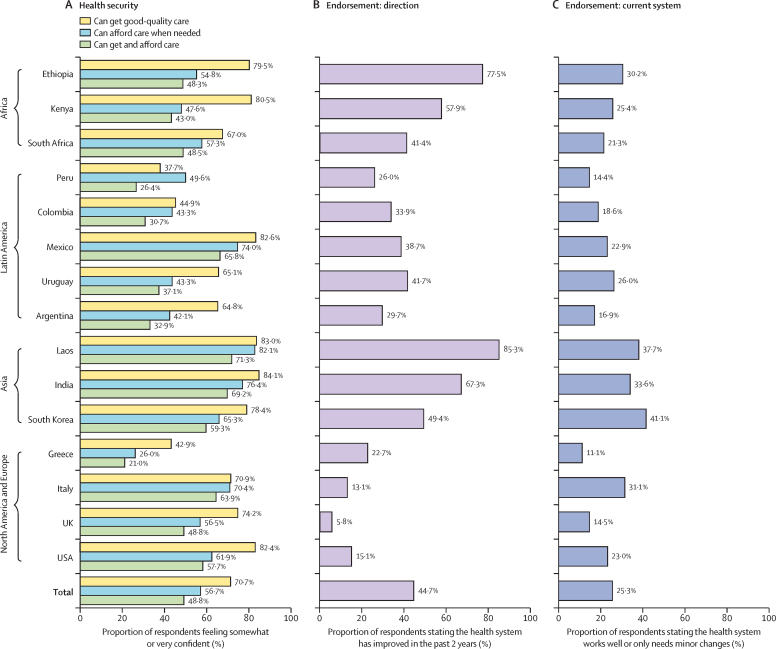
Descriptive findings on health system confidence

**Figure 3:**
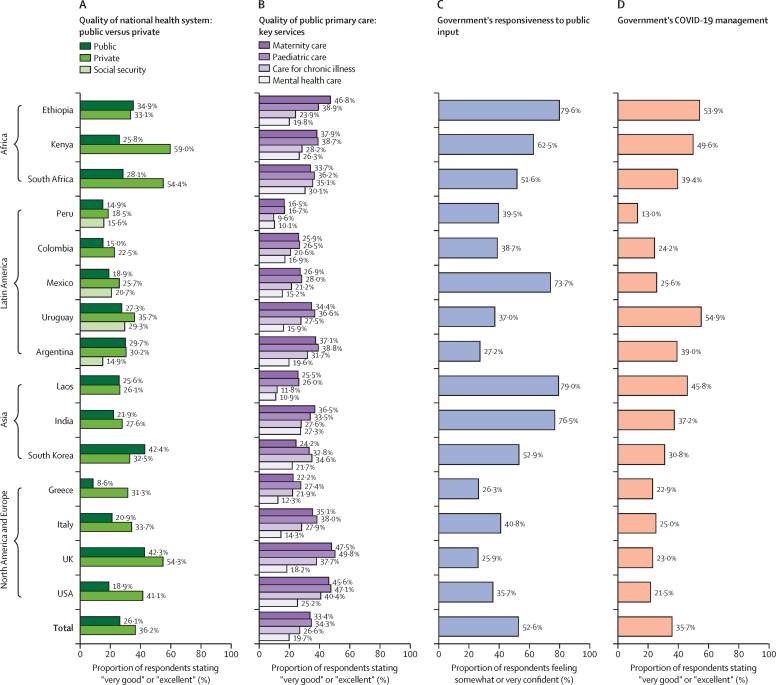
Descriptive findings on health system quality

Results from descriptive analyses of the confidence and quality measures across all countries are displayed in Figure 2, Figure 3. Confidence in getting good-quality care when needed was 70·7% on average across countries, with the greatest confidence in India (84·1%), followed by Laos (83·0%), Mexico (82·6%), the USA (82·4%), and Kenya (80·5%; figure 2A). By contrast, only 37·7% of respondents in Peru reported confidence in the ability to get good-quality care when needed, which could be due to government dysfunction or mismanagement during the COVID-19 pandemic. In all countries, fewer people reported that they were somewhat or very confident that they could afford care if they became very sick than reported getting good-quality care if they became very sick (figure 2A). Within global regions, people in Kenya and Ethiopia had lower confidence in affording care than people in South Africa; similarly, people in Argentina, Colombia, and Uruguay had lower confidence than people in Peru and Mexico. Confidence in both getting good-quality care and affording care were lowest in Peru (26·4%), Colombia (30·7%), and Greece (21·0%).

More than half of respondents believed that their health system had improved in the past 2 years in Kenya (57·9%), Ethiopia (77·5%), India (67·3%), and Laos (85·3%; figure 2B). Countries with the fewest respondents reporting that their health system was getting better were Italy (13·1%), the UK (5·8%), and the USA (15·1%). On average, only 25·3% across all countries reported that their system works well, and that only minor changes are necessary, with most people believing major changes are needed or that their health system needs to be rebuilt (figure 2C). Overall, although most respondents saw improvement, most wanted major change, and very few felt secure in their health system.

The percentage of respondents that reported “very good” or “excellent” for the quality of their country's public health system, private system, and social security or other system is shown in figure 3. The quality of maternity, paediatric, chronic conditions, and mental health care is also indicated. In all countries, public primary care for chronic conditions and mental health had the worst ratings, with mental health care routinely rated worst of all (19·7% rated “very good” or “excellent” across all countries). Public primary care for women and children were rated much higher, but less than 50% of respondents reported “very good” or “excellent” care in all countries (figure 3B). Quality of the national private system was rated higher than the national public system in almost all countries, with the greatest gap between the private and public system in South Africa (26·3%) and Kenya (33·2%; figure 3A).

## Factors associated with confidence and quality

**Figure 4:**
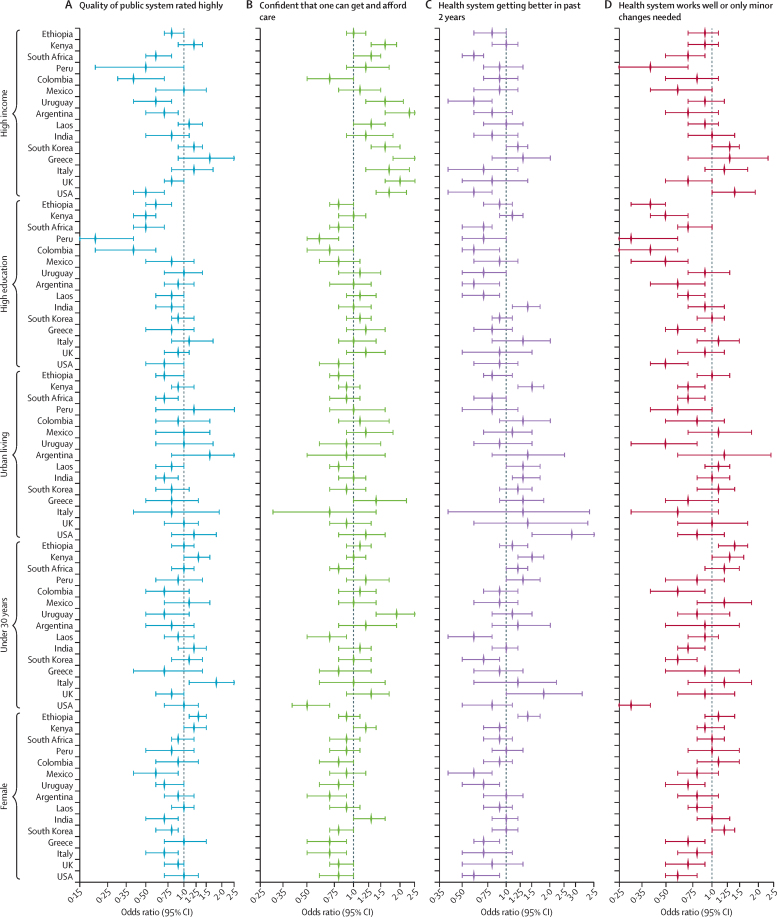
Factors associated with confidence and quality

The results of individual-country logistic regressions are reported in figure 4, in which the outcomes were quality and confidence measures, and the covariates were demographic factors of interest (ie, income, education level, urbanicity, age, and gender, controlling for self-rated health). In general, for most countries, higher income, higher education levels, and female gender were inversely associated with a high rating of the national public system (figure 4A). Similarly, female gender was inversely associated with confidence in getting and affording care if very sick in almost all countries (figure 4B). More educated people, wealthier people, and women were also less likely to report that their health system improved in the past 2 years, and that the health system only needs minor changes, compared with less educated people, those on lower incomes, and males (figure 4C, D). Additional sensitivity analyses for South Korea and the USA found that female gender was also inversely associated with quality ratings of the private system (appendix 2 p 10).

Results from the multicountry logistic regressions exploring factors associated with confidence measures are displayed in the table. Health system users were more likely to report that they could get and afford care if needed than non-users. People who were very confident that the government considers their opinion when making decisions about the health system were more than six times more likely to be very confident they can both get and afford care, almost four times more likely to believe their health system is getting better, and more than two times more likely to think the health system works well and only minor changes are needed.

**Table:** Pooled logistic regression results for association between confidence and individual, health system, and political factors

		**Respondent is somewhat or very confident that they can access and afford good-quality care when sick**	**Health system getting better in the past 2 years**	**Health system works well; only minor changes are needed**
Lower income		0·70*** (0·65–0·75)	1·04 (0·96–1·12)	1·08 (0·99–1·17)
Under 30 years		0·96 (0·89–1·03)	1·09* (1·00–1·18)	0·89** (0·82–0·97)
Post-secondary or higher education		1·10** (1·03–1·18)	0·98 (0·91–1·06)	0·76*** (0·71–0·82)
Urban living		0·98 (0·90–1·06)	1·27*** (1·16–1·39)	0·91* (0·83–1·00)
Self-rated health is very good or excellent		1·15*** (1·07–1·24)	0·90** (0·84–0·98)	1·02 (0·95–1·11)
Has chronic illness		0·91* (0·84–0·98)	0·93 (0·86–1·01)	1·02 (0·94–1·11)
Female		0·90** (0·85–0·96)	0·90** (0·84–0·97)	0·94 (0·88–1·01)
Has an unmet need for care		0·68*** (0·62–0·75)	0·86** (0·77–0·95)	0·81*** (0·72–0·91)
Health system user		1·18*** (1·09–1·29)	1·00 (0·91–1·09)	1·07 (0·98–1·17)
Quality of public system (reference: poor**)**				
	Fair	1·74*** (1·55–1·96)	2·34*** (2·06–2·66)	1·66*** (1·41–1·97)
	Good	2·69*** (2·39–3·03)	3·32*** (2·91–3·79)	2·58*** (2·18–3·05)
	Very good	3·50*** (3·05–4·02)	4·67*** (4·02–5·44)	4·25*** (3·55–5·08)
	Excellent	4·03*** (3·40–4·79)	5·66*** (4·71–6·80)	5·03*** (4·12–6·14)
Quality of private system (reference: poor**)**				
	Fair	1·28** (1·09–1·51)	1·27* (1·06–1·52)	0·9 (0·75–1·10)
	Good	1·69*** (1·44–1·98)	1·41*** (1·18–1·69)	0·91 (0·76–1·10)
	Very good	1·99*** (1·69–2·34)	1·49*** (1·24–1·79)	0·99 (0·82–1·19)
	Excellent	2·12*** (1·78–2·54)	1·45*** (1·18–1·78)	1·1 (0·90–1·36)
COVID-19 management (reference: poor**)**				
	Fair	0·99 (0·88–1·11)	1·53*** (1·34–1·75)	1·02 (0·89–1·18)
	Good	1·14* (1·02–1·28)	2·01*** (1·76–2·29)	0·96 (0·83–1·10)
	Very good	1·12 (0·99–1·26)	2·62*** (2·28–3·01)	1·13 (0·98–1·30)
	Excellent	1·05 (0·92–1·20)	3·54*** (3·04–4·12)	1·49*** (1·27–1·73)
Government considers opinion, (reference: not at all confident)				
	Not too confident	1·45*** (1·32–1·60)	1·57*** (1·41–1·75)	1·43*** (1·26–1·62)
	Somewhat confident	4·00*** (3·63–4·41)	2·19*** (1·97–2·44)	1·94*** (1·71–2·19)
	Very confident	6·55*** (5·81–7·38)	3·77*** (3·33–4·28)	2·52*** (2·19–2·90)
Country, (reference: Ethiopia)				
	Kenya	1·18* (1·02–1·36)	0·51*** (0·43–0·60)	0·93 (0·79–1·10)
	South Africa	1·23** (1·06–1·42)	0·23*** (0·20–0·27)	0·9 (0·76–1·07)
	Peru	0·71*** (0·60–0·85)	0·20*** (0·17–0·24)	0·73** (0·58–0·91)
	Colombia	0·80* (0·68–0·95)	0·22*** (0·19–0·26)	1·04 (0·85–1·27)
	Mexico	2·54*** (2·11–3·05)	0·19*** (0·16–0·23)	0·85 (0·69–1·05)
	Uruguay	0·82* (0·69–0·98)	0·18*** (0·15–0·22)	1·34** (1·12–1·61)
	Argentina	0·89 (0·74–1·07)	0·16*** (0·14–0·20)	0·87 (0·70–1·08)
	Laos	2·56*** (2·20–2·97)	1·69*** (1·42–2·02)	1·99*** (1·71–2·32)
	India	2·76*** (2·34–3·24)	0·88 (0·75–1·05)	1·59*** (1·34–1·88)
	South Korea	1·62*** (1·40–1·87)	0·30*** (0·26–0·35)	2·79*** (2·40–3·25)
	Greece	0·51*** (0·43–0·61)	0·20*** (0·17–0·23)	0·82 (0·66–1·00)
	Italy	3·88*** (3·22–4·68)	0·05*** (0·04–0·07)	2·43*** (2·01–2·92)
	UK	1·74*** (1·47–2·07)	0·02*** (0·01–0·02)	0·70** (0·57–0·87)
	USA	2·48*** (2·12–2·90)	0·07*** (0·06–0·09)	1·46*** (1·23–1·74)
	N	21 647	21 691	21 735

Exponentiated coefficients; 95% confidence intervals in parentheses. Results from three multicountry logistic regressions where the outcomes were (1) reporting “very confident” or “somewhat confident” for being able to get and afford good-quality care if very sick (*vs* “not too confident” or “not at all confident” on either item), (2) reporting that the health system has improved in the past 2 years (*vs* staying the same or getting worse), and (3) reporting that the health system works well and only minor changes are necessary (*vs* major changes being needed, or the system needing to be rebuilt). Covariates included in all models were: poor (low income=1, and highest or middle income=0), age (younger than 30 years=1, older than 30 years=0), post-secondary or higher education complete (post-secondary education or higher education complete=1; none, primary, or secondary education complete=0), urban living (urban=1, rural=0), self-rated health (excellent or very good=1; good, fair, or poor=0), has chronic illness (yes=1, no=0), female (female=1, male=0), has an unmet need for care (yes=1, no=0), health system user (one or more visits in past 12 months=1, 0 visits in past 12 months=0), quality of public system (excellent=4, very good=3, good=2, fair=1, poor=0), quality of private system (excellent=4, very good=3, good=2, fair=1, poor=0), COVID-19 management (excellent=4, very good=3, good=2, fair=1, poor=0), government considers the respondent's opinion in improving health systems (very confident=3, somewhat confident=2, not too confident=1, not at all confident=0), and country fixed effects. Argentina includes data only from the Province of Mendoza.

* p<0·05,

** p<0·01,

*** p<0·001.

## Discussion

In this 15-country population assessment of health system performance 2 years after the start of the COVID-19 pandemic, we found overall low confidence in the health system across countries and income levels. Marked differences are apparent across regions and demographic characteristics of respondents. We make six observations.

First, people's sense of security in the health system was relatively low across the countries assessed. Less than half of respondents on average across countries reported being somewhat or very confident that they could both get and afford the care they need if they became very sick. These combined indicators reflect people's views of universal health coverage—a policy priority in all the studied countries that is aimed at improving health and providing a financial safety net.^42^ These results are perhaps expected in countries with low health spending that are just beginning to invest in universal health coverage, but they are more concerning in high-income countries that have attained universal health coverage (ie, providing an extensive benefit package financed through prepayment to the population). For example, in high-income countries with universal health insurance, such as the UK, Italy, Greece, and South Korea, only 21–60% of people reported being able to get and afford good care, suggesting that insurance does not cover desired services or that covered services are not meeting people's quality expectations (which, in some cases, might not be realistic). The lowest level of health security was in Greece, where austerity measures in the past decade compounded already low funding for the public health system, with concomitant increases in unmet need.^43^, ^44^, ^45^ This low health security is consistent with historically low trust in social and political institutions in Greece compared with other European countries.^46^ Although comparison data are not available for most countries, since 2013 a 10 percentage point decline in confidence in getting good care in Colombia has been observed, while Mexico's confidence level has remained stable.^37^ Perceptions of public systems and, in turn, confidence in some high-income countries could have eroded over time as the provision of private care expanded and people's experience of poor customer service or low government responsiveness increased, even in the context of technically good care—especially in systems overwhelmed by COVID-19.^47^, ^48^

With the exception of Mexico, Latin American respondents had some of the lowest reported health security, whereas African respondents reported higher health security and endorsement. This finding does not comport with low health system spending and quality of care challenges in sub-Saharan Africa.^2^, ^49^, ^50^ Higher confidence in sub-Saharan Africa could be due to highly visible recent investments in health systems (eg, large increases in the number of health facilities) that were made from a lower starting point than in high-income countries.^51^ Rapid health investments have been linked to population trust in Nepal and China.^41^, ^52^ Respondents from Armenia, Belarus, Georgia, Kazakhstan, Kyrgyzstan, Moldova, Russia, Ukraine, and Azerbaijan (ie, countries that underwent large reforms) had increased satisfaction over time,^22^ which is in contrast to a general stagnation in health system development in Latin America.^53^, ^54^ Latin America has experienced a series of political and economic shocks in the past three decades, which have undermined public confidence in government institutions in many countries.^14^, ^20^ Low expectations of the health system, young median age, and different prevailing disease burdens (ie, fewer severe chronic conditions requiring advanced care) could also promote increased confidence in low-income regions. With economic growth and rising education levels, population expectations are likely to rise, resulting in decreased satisfaction over time.^21^, ^41^

Second, and consistent with these lower expectations, the majority of respondents in the lowest income countries (ie, Ethiopia, Laos, and Kenya) reported that systems were improving in the past 2 years, while only about a third agreed in middle-income countries (Argentina, South Africa, Peru, Colombia, and Uruguay). The lowest ratings were in the UK, followed by Italy and the USA. In addition to the pace of change in health system improvements, this assessment is probably influenced by how these countries fared in the COVID-19 pandemic in terms of cases, mortality rates, and social and health system disruptions.^55^, ^56^ For example, among the three European countries sampled, Greece fared better on this indicator than Italy and the UK, consistent with its lower COVID-19 mortality rates.^57^ In Latin America, countries' public distrust in government predated the pandemic but was probably exacerbated by the intensity of COVID-19 in the region. Local political and social dynamics probably had an important role in Peru, which had high COVID-19 mortality, multiple political upheavals in the past 2 years, and some of the lowest confidence ratings.^58^, ^59^ By contrast, in Kenya and Ethiopia, widely publicised health insurance expansion and increased visibility of health leaders during the COVID-19 pandemic could have promoted optimism in the health system, together with relatively fewer deaths and reported cases.

Overall, a minority of respondents in the study countries (11·1–41·1%) endorsed the current health system as is or with only minor changes. The highest endorsement was in South Korea, where the past two government administrations have pursued well publicised expansions of insurance coverage, and the lowest was in Greece, where negative sentiment could be linked to past austerity measures.^46^ Compared with past results from countries of similar income levels, overall endorsement appears to have decreased.^2^, ^41^ For study countries with available past data, endorsement was similar in Mexico, Kenya, South Africa, and India to what it was in 2017, and declined in Argentina from 29% to 17%.^30^ Endorsement fell substantially in the UK from 63% in 2013 to 15% in 2023.^2^

Third, country-level regression analysis showed that, after adjusting for other demographics, wealthier respondents reported higher confidence that they can get and afford care if sick compared with those on lower incomes. In half of the study countries, women had lower confidence than men. The reason for the lower ratings by women is unclear, possibly reflecting lower confidence in being able to extract good quality care, or having fewer resources to seek good care. Women use health care more frequently and for different conditions, and in some settings they have more negative experiences than men.^60^, ^61^ Wealthier, more educated, and female respondents reported a lower quality of public health systems. Post-secondary education was strongly negatively associated with health system endorsement (ie, the system improving in the past 2 years and the system working well now) in most countries. Women in the USA had some of the lowest endorsement ratings—possibly due to the extensively publicised erosion of abortion rights in the past year. People younger than 30 years were substantially less likely to endorse the current system in the USA, Colombia, Laos, and South Korea compared with people older than 30 years. This reduced support for the health system among key demographic groups suggests a fragmentation of social solidarity that is a prerequisite for pooling funds for a national health service^22^, ^62^

Fourth, quality ratings of public primary care services favoured maternal and child health over chronic disease and mental health, particularly in low-income countries. Other studies found low levels of support (<50%) for primary care in low-income and middle-income countries.^63^ Across all countries, participants in our study rated mental health care at or near the bottom of all services, with fewer than one in five respondents giving a high rating. This low rating is in stark contrast to the size of the epidemiological burden presenting to primary care in most countries, led by chronic conditions (eg, hypertension and diabetes) and common mental health disorders (eg, depression and anxiety).^64^, ^65^ Mental health care is a highly neglected service in health systems in low-income and middle-income countries and is under growing strain in high-income countries.^66^, ^67^ Stigma among the population and health workers also contributes to low access and quality.^68^, ^69^

Fifth, across all countries studied, only a quarter of respondents rated the overall quality of the government health system as “very good” or “excellent”. Ratings for private and government health systems were very similar in countries with low penetration of private health care (eg, Ethiopia, Laos, and Peru) or in systems where government or social security authorities provide care (eg, Colombia and Uruguay).^70^ In countries where the two systems exist independently, private care received substantially higher ratings than public care (eg, the USA, Greece, Italy, Kenya, and South Africa). Notably, Greece had the largest gap in perceived quality between public and private systems, possibly due to historically low spending on the public health-care system and austerity-driven reductions, with its current spending well below the EU average.^71^, ^72^ In most countries, private health systems are substantially better resourced than public systems, although quality of care is not always higher.^73^, ^74^, ^75^, ^76^ Health system ratings are not routinely obtained in surveys and cut points for positive ratings differ, but broadly similar levels of satisfaction with health systems have been noted in studies.^51^, ^77^, ^78^

Finally, in pooled (all-country) regressions that included country fixed effects to capture local context, we found that health security was very strongly associated with government responsiveness (ie, public opinion being considered in health policies) and quality of the public health system. Quality of the public sector has previously been found to be associated with satisfaction.^22^ Agreement that the system was improving over the past 2 years was most strongly associated with quality of the public health system, COVID-19 management, and government responsiveness. Endorsement was most strongly influenced by quality of the public health system and responsiveness. Higher rates of COVID-19 infections and mortality have been associated with lower trust in the health system in other settings, including Brazil.^79^ Arakelyan and colleagues found that trust in health systems is determined by consistent and positive exposure to health services, but also that historical narratives and societal changes shape both trust and care-seeking.^4^, ^80^ Rockers and colleagues identified an association between health system performance factors (eg, quality, responsiveness, fair treatment, and financial risk protection) and trust in public health institutions in 38 low-income and middle-income countries.^13^ Associations with demographic factors were similar to those identified in the literature, with younger and more educated respondents being less likely to endorse health systems than their older and less educated peers.^22^, ^81^

Our analyses are subject to several limitations. First, people's perceptions of health care are influenced by various factors, including education, personality, health, cultural and social factors, and individual preferences that might not have been fully captured in the survey.^29^ We adjust for these factors in some of our analyses but not in the descriptive results, as the unadjusted (population-weighted) data best indicate prevailing opinion in the country. Second, information asymmetry between providers and patients might prevent patients from correctly judging technical quality. The majority of items in the PVS relate to user experience or perceived performance, for which people are the best source of information through their own use or reports from family and friends.^82^ User-reported satisfaction ratings aligned with several important quality outcomes but they also reflect user factors that are not captured here.^78^, ^82^, ^83^ Third, low prevailing health system quality in some countries and low education and health literacy might reduce people's health-care expectations, inflating confidence and endorsement and complicating cross-country comparison.^84^ We interpret the findings here with regard to those differences and note country-specific contexts that could influence results. Fourth, people in the USA have little experience with publicly provided health care and might therefore provide a less informed rating than respondents in other countries. Finally, study results are likely to be highly influenced by the COVID-19 pandemic and attendant health system shocks. The PVS was designed to be repeated every 2 years.

Our study has several strengths, most notably the use of comparable measures for undermeasured but policy-relevant constructs, such as health security, endorsement, and system quality across a wide range of countries. The PVS captures previously undermeasured aspects of health system performance, including confidence in the health system, and should be integrated into the routine measurement of health system performance to promote accountability and improvement in health systems that aim to be people-centred.

Our findings have various policy implications. Countries should rethink their provision (and measurement) of quality mental health services, as the current population perception of quality probably reflects the reality of such care in many countries and could discourage people with needs from accessing care.^85^, ^86^ The divergent views of public and private health-care quality, along with low health system endorsement from more educated, wealthy, and younger respondents in many countries, signals a segmentation in support for public systems. This divide should be a call to action for governments to invest in publicly financed health care and to effectively communicate its value to the public. Governments and health authorities need to strengthen mechanisms for public input into health system design as this is linked to greater public confidence. Finally, the surprisingly low confidence in the health systems of middle-income and high-income countries should prompt a national dialogue about the future direction of health care, including societal funding trade-offs, policy innovation, and service delivery redesign, to maximise the value of health systems to users and non-users.

## Data sharing

Individual-level, de-identified data from the People's Voice Survey will be publicly available in mid-2024. Data will be available on the Harvard Dataverse (https://dataverse.harvard.edu). The survey instrument and data dictionary will be available upon publication.

## Declaration of interests

CA received funding from Merck that was used to conduct the People's Voice Survey in the USA, Mexico, Italy, and the UK. ECB received support for the manuscript from WHO Athens Quality of Care and Patient Safety Office Consultancy Contract. JB and SC received WHO support. EAO received support from the Wellcome Trust Senior Fellowships (number 224272) and the Wellcome Trust Kenya Major Overseas Programme (number 203077), consulting fees for serving on the AstraZeneca Vaccine and Immune Therapies Effectiveness Evidence Scientific Advisory Committee, and support for meetings and travel for TDR Scientific and Technical Advisory Committee meeting attendance supported by WHO. GSF received support for the manuscript provided by the Bill and Melinda Gates Foundation and the Swiss Agency for Development and Cooperation; grants or contracts through the US Centers for Disease Control and Prevention, the Association of State and Territorial Health Officials, and the National Institute of Aging and National Institutes of Health; and support for attending meetings and for travel through Dartmouth College.
