# Supplementary material for: Population confidence in the health system in 15 countries: results from the first round of the People's Voice Survey
Source: Lancet Glob Health. 2023 Dec 11;12(1):e100–11. doi: 10.1016/S2214-109X(23)00499-0 (PMC10716625; doi:10.1016/S2214-109X(23)00499-0)
Supplement: Supplementary appendix 1 [file mmc1.pdf]

THE LANCET
Global Health

Supplementary appendix 1

This appendix formed part of the original submission and has been peer reviewed. We post it as supplied by the authors.

Supplement to: Kruk ME, Kapoor NR, Lewis TP, et al. Population confidence in the health system in 15 countries: results from the first round of the People's Voice Survey. *Lancet Glob Health* 2023; published online Dec 11. https://doi.org/10.1016/S2214-109X(23)00499-0.

Appendix 1: Detailed Methodology for the People's Voice Survey

The People's Voice Survey (PVS) was developed using international best practices for survey research.[1,2] The content was guided by the *Lancet Global Health* Commission on High Quality Health Systems in the Sustainable Development Goals Era (HQSS Commission) conceptual framework with question wording, response options, and sequencing informed by reviews of prior surveys used in higher- and lower-income countries and by input from the PVS Global Development Group (GDG): health system academics, managers, policymakers, and health care users. Content validity was further tested through external peer review by health system experts in international organizations and survey methods specialists. The questionnaire was assessed for comprehension in most countries via cognitive interviews that included open-ended questions concerning key concepts. The instrument was translated into local languages by professional translators and pre-tests were conducted in all settings to refine question wording and local response options. The survey was piloted in each country by the study contractors and corrections were made by local research teams prior to mainstage data collection.

Survey mode, sampling, and weighting

In Wave 1 countries, the PVS was primarily delivered via telephone interview (Supplement B table 1). Where necessary to reach the population, we used web-based surveys (South Korea) or a stratified approach that included both telephone and web-based surveying (the United Kingdom and the United States). To ensure the data represented most of the adult population, we required a minimum population coverage level of 80% mobile and landline telephone ownership (Supplement B table 2). In settings with lower coverage, face-to-face (FTF), household-based surveys were required to fully represent the population, especially in rural areas.[3,4] In these settings, the PVS was implemented with a stratified approach that included telephone surveys in areas that had higher telephone ownership and face-to-face surveys of people in lower-ownership areas (Ethiopia and Kenya).

The PVS aims to obtain population sentiment about performance of the health system by estimating population proportions agreeing with a range of statements. A survey of 1,000 individuals selected at random will produce an estimate that is within a 3% margin of error of the population proportion 95% of the time. This is the case when the prevalence is 50%; smaller numbers are needed when prevalence is higher or lower. Thus, we used a minimum sample of 1,000 in all countries to obtain sufficient precision on all key measures of interest. Several of our samples were larger than this to permit some stratified analysis (e.g., by urban/rural) (Supplement B table 1).

When conducted via telephone interviewing, PVS respondents were selected through random-digit dialing (RDD) or a known-list sampling approach (Ethiopia, Argentina). In South Korea, the United Kingdom, and the United States, respondents were selected from probability-based online panels (K-panel in Korea, Kantar Public Voice in the United Kingdom, and the SSRS Opinion Panel in the United States). Multi-stage cluster sampling, which included selection of rural primary sampling units (PSUs) and selection of households using random walk, was used to identify participants for FTF interviews in Ethiopia and Kenya.

To correct for design effects, inverse probability of selection weights for telephone samples (based on the number of telephone numbers available per respondent) and probability-proportional-to-size weights for FTF samples were constructed by country. Post-stratification weights based on external population statistics were used to adjust the sample on variables of importance to the survey, including age, gender, region, and education where possible to reduce sampling biases (Supplement B table 1). Post-stratification weights were not required for South Korea, where a prospectively stratified sample ensured representativeness across relevant factors, or in Argentina, where a quota sampling approach was used based on gender, age, department, and insurance coverage. Weights were constructed using an iterative proportional fitting (raking) approach.[5]

Data collection partners were Ipsos (https://www.ipsos.com) and SSRS (https://ssrs.com) in all countries except South Korea where data was collected by Kstat (http://www.kstat.co.kr) and Laos where researchers conducted interviews themselves. Before data collection, data collection supervisors and individual interviewers received formal training on the PVS to ensure understanding of study goals, the purpose of each question in the survey, potential obstacles to obtaining answers, and possible respondent problems and how to address them. Training sessions were led by QuEST researchers and/or data collection partners with participation from the QuEST team. Written materials on the survey instrument were provided prior to fieldwork, including an annotated questionnaire with explanations and key term definitions. Multiple training strategies were used across countries, including detailed questionnaire walk-throughs and interview roleplay. Pre- and post-training tests were conducted in three countries. Interviewer performance and data quality was ensured through additional supervisory review of a random subset of interviews via parallel listening or review of recordings. Wave 1 data collection began on May 9, 2022 in Laos and ended on April 3, 2023 in India. To contextualize these data, we describe national, health system, and health outcome factors for each country at the time of data collection (Supplement B table 2).

Survey development and validation

Questionnaire creation (Assessment of content validity)

There were three stages to developing the PVS questionnaire and ensuring content validity, defined as the extent to which the survey includes all required items to measure constructs of interest.[6] First, we developed the PVS instrument based on the High Quality Health Systems Framework established by the HQSS Commission. The framework has been widely cited in the health systems literature and ensures the survey is grounded in latest theory.[7] The PVS framework includes key domains of health system quality, including care processes and quality outcomes that matter most to people (Supplement B figure 1). Table 3 presents PVS domains and key indicators corresponding to each section of the framework.

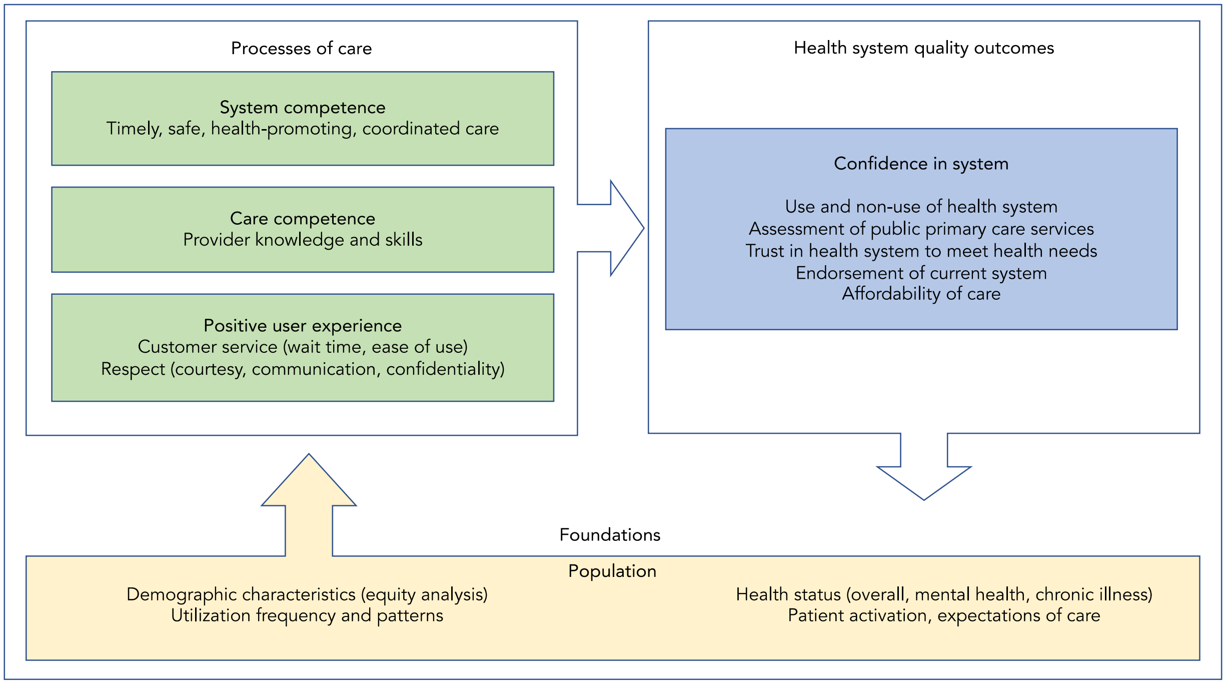

Supplement B figure 1: People's Voice Survey Framework

Notes: People care about outcomes beyond good health, which include trusting that the system can meet their needs, confidence that they can afford services, and endorsement of health system performance. These perceptions are informed by processes of care, including system competence (e.g., whether the health system provides coordinated, easy-to-use care integrated across platforms), care competence (e.g., provision of high-quality care from knowledgeable, high-skilled providers), and user experience (e.g., good customer service and respect). These processes and outcomes are underpinned by the foundations of the health system, including health status, demographic characteristics, patient activation, and expectations of care.

Supplement B table 3: Domains and key indicators from the People's Voice Survey

**Foundations (population factors)**
• Health<br>○ Health status<br>○ Mental health status<br>○ Chronic illness status<br>• Patient activation/empowerment<br>○ Level of activation<br>○ % of population who can bring up concerns to provider<br>○ % of population with low expectations<br>• Health care use<br>○ % of population with a type of insurance<br>○ % of population w/with usual source of care by facility ownership and level<br>○ Reasons for selecting usual source of care<br>○ Total visits, visits by facility, and type of visit (covid, virtual, home, inpatient)<br>○ Number of facilities used in past 12 months
**Processes of care**

• Care people need<br>○ Mental health service use<br>○ Respondents with chronic disease who have usual source of care<br>○ Respondents who needed but did not use health services by health status<br>○ Reasons respondents did not use care<br>• Health system competence<br>○ % of population who had recent screening (blood pressure, mammogram, cervical cancer, eyes, teeth, cholesterol)<br>• Health care quality<br>○ Quality rating of usual source of care<br>○ % of population who experienced a medical mistake in past 12 months<br>○ % of population who experienced discrimination in past 12 months<br>○ Quality of last health care visit: Overall, technical, and interpersonal quality; Service readiness; Wait time at facility; Time spent with clinician<br>○ Endorsement of usual source of care clinic
**Health system quality outcomes**
• Confidence in public primary care<br>○ Confidence in services for pregnant women, sick children, chronic illness, mental health<br>• Trust in the health system<br>○ Confidence in ability to get needed care<br>○ Affordability of needed care<br>○ Health system ratings (public, private, NGO)<br>○ Whether people have a say in the system<br>○ Trend in health system performance<br>○ Need for reform / health system "endorsement"<br>○ Rating of government COVID-19 management

Second, we conducted a collaborative, multistage process to develop and validate the PVS (Supplement B figure 2). To guide this process, we assembled a diverse development group comprised of 30 health system experts from 18 high-, middle-, and low-income countries. GDG members were expert in areas such as survey design, health system quality, health care preferences, patient safety, national health system organization and financing, maternal and child health, chronic diseases, mental health, primary health care, and health system policymaking. The group assessed all aspects of the survey in development, including aims, methodology, and policy opportunities, with a special focus on content, including key domains overlooked in existing surveys.

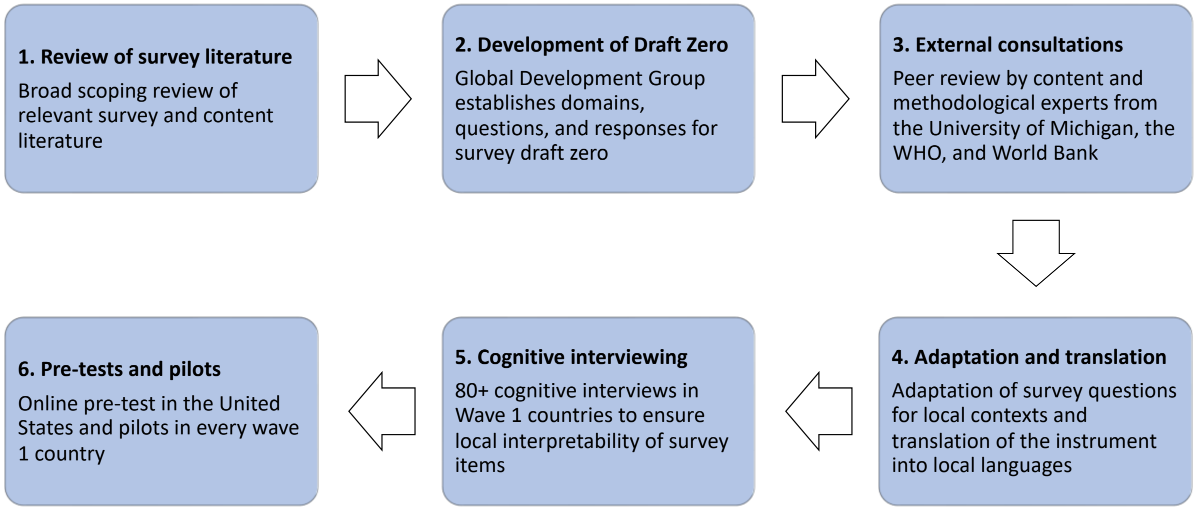

Supplement B figure 2: Development and validation steps of the People's Voice Survey

Development group researchers conducted a broad scoping review of recent survey literature and relevant survey tools to identify questions and response options used to measure key survey domains. Where possible, we used survey items already asked in validated and/or widely-used surveys. The GDG generated survey aims and priorities, critically appraised survey domains and items for value, clarity, and relevance, and assessed survey construction. The group met regularly over the course of 18 months in an iterative process to help ensure content validity and co-produce a "draft zero" instrument.

Third, the GDG sought external consultation on both methodology and content of the survey. This process included peer review by survey experts from the University of Michigan and content and measurement experts from academic and multilateral organizations, including the World Health Organization (WHO) and World Bank. Reviewers assessed for best practices in survey design and delivery, such as question formulation and flow, and completeness and relevance of survey content. This feedback informed subsequent drafts of the instrument and further bolstered content validity.

Survey adaptation (Assessment of face validity and content equivalence)

In Wave 1 countries, the survey instrument underwent a collaborative adaptation by local researchers and/or policymakers, including cognitive interviewing, to ensure the instrument would be locally applicable, interpretable, and comprehensive. This process was intended to maintain content equivalence by ensuring cross-national comparability, clarity, and local relevance of questions in each setting. We defined content equivalence as whether survey items were understood consistently in different settings.[8]

PVS implementers in each setting identified the minimum set of survey languages necessary to reach most of the national population based on recent, nationally-representative surveys. When possible, we used a team translation approach; at minimum, translation and back-translation were

performed by qualified translators and closely reviewed by QuEST researchers to ensure cross-context equivalence in each language.

We conducted cognitive interviews to bolster both content equivalence and establish face validity, defined as whether items represent constructs "on their face."[9] Cognitive interviewing assessed whether survey items fulfilled their expected purpose (e.g., correctly understood by participants, no problems with wording, no difficulties with response options) and helped to identify any concerns about survey flow or length.[10] The GDG conducted cognitive interviews in multiple settings using a standardized, semi-structured interview guide. We used a criterion sampling approach to deliberately obtain variability by age, language, health system use, and other factors. Interviews were recorded and analyzed to identify problem areas. QuEST researchers conducted over 80 cognitive interviews in eleven countries and seven languages. Results helped to establish face validity for the instrument and highlighted multiple areas for improvement, including cutting lengthy items and simplifying questions with high cognitive burden. For example, feedback from cognitive debriefings supported the use of a single question on self-rated health instead of a multi-question scale across countries. In Colombia, Peru, and Uruguay, wording changes to questions and response options were needed to clarify types of insurance coverage and levels of care. In some countries, socio-demographic questions were adapted to local sensitivities.

Pre-testing and piloting (Assessment of criterion and construct validity)

We additionally conducted pretests in the United States to assess survey length and coherence. The survey was self-administered in English and Spanish to 200 respondents over age 18 years from an online panel with varying education levels and ages, as well as a small group of respondents over age 65 years who were expected to have higher health care utilization than their younger counterparts. Overall, respondents completed the survey in the expected time and found survey questions easy to answer. We made several edits based on these experiences, including removal of multiple options that were challenging for respondents.

Finally, we conducted pilot surveys in one or more languages in each country. We checked the quality of the response data, including completeness of responses, response rates, internal consistency (e.g., consistent responses on utilization patterns and number of visits), and distribution of responses (e.g., too many "I don't know" responses). In response to the analysis of pilot data, we adjusted response options due to overuse of "other" categories, verified certain responses with external population data (e.g., COVID-19 vaccination rates), added interviewer instructions to guide respondents in areas of potential confusion, and updated relevant interviewer training materials.

We used these pre-test and pilot data to assess criterion validity, defined as the relationship between survey data and an existing criterion or "gold standard."[13] While there is no single gold standard measure by which to assess the PVS, we evaluated whether PVS results comported with data from other large, widely-used surveys (concurrent validity) and whether PVS items were predictive of other relevant items in our survey (predictive validity).[11] For example, we measured the relationship between respondent education level and rating of the survey vignette of objectively poor quality of care and found that respondents who reported completing primary

school or less were more likely to rate poor quality care as "excellent" than those who completed secondary school or higher, as found elsewhere.[12] We also found, for example, that rates of chronic illness, preventive screenings, and COVID-19 vaccination in the PVS were similar to national surveys in each country and to data reported by multi-national organizations. For predictive validity, we found that countries with higher rates of private health system use also had higher ratings of the private health system and that respondents with chronic illness had more health care contacts.

We also investigated convergent and discriminant validity, two forms of construct validity that assess whether survey items that measure the same or related constructs correlate or diverge as expected.[14] For example, we compared ratings of care quality at the last health care visit in public health facilities and found they were closely associated with ratings of the public health system overall. We also examined whether respondents were discriminating between similar, but distinct questions regarding overall assessment of the health system. For example, we asked respondents their opinion on the health system trajectory (getting better, staying the same, getting worse) and on health system need for reform (whether it needs a minor, major, or complete reform). Though asked consecutively, these similar questions were often answered differently by respondents (e.g., system is getting better but needs a major reform), indicating respondents are successfully differentiating underlying constructs between each item.

Supplementary references

1 OECD. Good Practices in Survey Design Step-by-Step. Paris: OECD, 2012.

2 Üstun TB, Chatterji S, Mechbal A, Murray C. Quality assurance in surveys: standards, guidelines and procedures. *Household sample surveys in developing and transition countries* 2005; **2005**: 199–230.

3 Gourlay S, Kilic T, Martuscelli A, Wollburg P, Zezza A. Viewpoint: High-frequency phone surveys on COVID-19: Good practices, open questions. *Food Policy* 2021; **105**: 102153.

4 Kastelic KH, Eckman S, Kastelic JG, Mcgee KR. High frequency mobile phone surveys of households to assess the impacts of COVID-19 (Vol. 2): guidelines on sampling design. 2020.

5 Kolenikov S. Calibrating Survey Data using Iterative Proportional Fitting (Raking). *The Stata Journal* 2014; **14**: 22–59.

6 Rossiter JR. Content Validity of Measures of Abstract Constructs in Management and Organizational Research. *British Journal of Management* 2008; **19**: 380–8.

7 Kruk ME, Gage AD, Arsenault C, *et al.* High-quality health systems in the Sustainable Development Goals era: time for a revolution. *The Lancet Global Health* 2018; **6**: e1196–252.

8 Frongillo EA, Baranowski T, Subar AF, Tooze JA, Kirkpatrick SI. Establishing Validity and Cross-Context Equivalence of Measures and Indicators. *Journal of the Academy of Nutrition and Dietetics* 2019; **119**: 1817–30.

9 Fitzner K. Reliability and Validity A Quick Review. *Diabetes Educ* 2007; **33**: 775–80.

10 Scott K, Ummer O, LeFevre AE. The devil is in the detail: reflections on the value and application of cognitive interviewing to strengthen quantitative surveys in global health. *Health Policy and Planning* 2021; **36**: 982–95.

11 Cronbach LJ, Meehl PE. Construct validity in psychological tests. *Psychological bulletin* 1955; **52**: 281.

12 Roder-DeWan S, Gage AD, Hirschhorn LR, *et al.* Expectations of healthcare quality: A cross-sectional study of internet users in 12 low- and middle-income countries. *PLOS Medicine* 2019; **16**: e1002879.

13 Drost EA. Validity and reliability in social science research. *Education Research and Perspectives* 2011; **38**: 105–23.

14 Colliver JA, Conlee MJ, Verhulst SJ. From test validity to construct validity … and back? *Medical Education* 2012; **46**: 366–71.

Supplement B table 1: Survey methodology for each Wave 1 country

Country	Average survey length by mode (minutes)	Fieldwork dates	Survey languages	Data collection partner	Response rate (%)	Sample sizes	Sampling frame (mode)	Weighting variables
**Colombia (CO)**	CATI: 33.58	7/7/22 – 8/23/22	Spanish	Ipsos	13%	1,237	RDD (CATI)	Age, gender, region, education
**Ethiopia (ET)**	CATI: 24.12<br><br>F2F: 24.11	8/16/22 –11/4/22	Afan Oromo, Amharic, Tigrigna, Somali	Ipsos	32% (CATI)<br><br>58% (F2F)	2,445 (CATI)<br><br>334 (FTF)	Known-list sampling (CATI) and multi-stage clustered design (FTF)	Age, gender, region, education
**India (IN)**	CATI: 23.17	2/20/23 - 4/3/23	English, Hindi, Marathi, Tamil, Telegu, Bengali, Assamese, Gujarati, Kannada	Ipsos	8%	2,004	RDD (CATI)	Age, gender, region, education
**Kenya (KE)**	CATI: 19.64<br><br>FTF: 25.52	8/17/22 – 10/08/22	Swahili, English	Ipsos	35% (CATI)<br><br>84% (F2F)	2,006 (CATI)<br><br>299 (FTF)	RDD (CATI) and multi-stage clustered design (FTF)	Age, gender, region, education
**Peru (PE)**	CATI: 24.69	7/05/22 – 9/15/22	Spanish	Ipsos	6%	1,255	RDD (CATI)	Age, gender, region (Lima and other), education
**South Africa (ZA)**	CATI: 26.61	10/20/22 - 1/20/23	English, Afrikaans, isiXhosa, isiZulu, Sepedi, Sesotho, Setswana	Ipsos	10%	2,036	RDD (CATI)	Age, gender, region, education
**Uruguay (UY)**	CATI: 18.68	7/12/22 – 9/19/22	Spanish	Ipsos	8%	1,237	RDD (CATI)	Age, gender, region (2 largest regions and other), education
**Laos (LA)**	CATI: 22.26	5/9/22 – 8/19/22	Lao, Hmong, Khmou	In-house	18%	2,007	RDD (CATI)	Age, gender, region, education, urban/rural

**United States (US)**	CAWI: 16.37<br><br>CATI: 27.63	12/14/22 - 1/23/23	English, Spanish	SSRS	2%	50 (CATI)<br><br>1,450 (CAWI)	Online probability panel	Age, gender, race/ethnicity, education, census region, population density, civic engagement, internet use frequency, voter registration status, party ID, and religious affiliation
**Mexico (MX)**	CATI: 22.67	12/21/22 - 1/31/23	Spanish	SSRS	3%	1,002	RDD (CATI)	Age, gender, education
**Italy (IT)**	CATI: 22.71	12/16/22 - 1/20/23	Italian	SSRS	5%	1,001	RDD (CATI)	Age, gender, education
**South Korea (KR)**	CAWI: 19.72	2/10/23 - 2/16/23	Korean	Kstat	5%	2,000	Online probability panel	Age, gender, region, education (through stratified sampling)
**Argentina (AR)**	CATI: N/A	9/22/22 - 1/16/22	Spanish	Ipsos	4%	1,190	Known-list sampling	Age, insurance coverage, region
**United Kingdom (GB)**	CATI: 29.9<br><br>CAWI: 17.2	3/17/23 - 3/29/23	English	SSRS	4%	92 (CATI)<br><br>1,585 (CAWI)	Online probability panel	Age, gender, and education
**Greece (GR)**	CATI: 18.03	5/17/23-7/16/23	Greek	Ipsos	18%	2,015	RDD (CATI)	Age, gender, region, education

Notes: RDD = Random digit dialing; CATI = Computer-assisted telephone interviewing; CAWI = Computer-assisted web interviewing; FTF = face-to-face. The response rate (%) for CATI surveys is the American Association for Public Opinion Research (AAPOR) Response Rate 3 (RR3). Multi-stage clustered sampling for FTF interviews included selecting rural primary sampling units (PSUs) and random walk sampling to select households.

Supplement B table 2: Wave 1 country characteristics at time of data collection

General indicators	Argentina	Ethiopia	Kenya	South Africa	Peru	Colombia	Mexico	Uruguay	India	South Korea	Laos	Italy	United Kingdom	United States	Greece
Population (thousands)[1]	45,808.75	120,283.03	53,005.61	59,392.25	33,715.47	51,516.56	126,705.14	3,426.26	1,407,563.84	51,744.88	7,425.06	59,109.67	67,326.57	331,893.74	10,566.531
GNI per capita, PPP (current international $)[1]	23,170	2,530	5,130	14,340	12,780	16,540	19,740	22,950	7,130	47,770	8,100	46,450	50,540	70,480	36,600
Urban population (% total population)[1]	92	22.2	28.5	67.8	78.5	81.7	81	95.6	35.4	81.4	36.9	71.3	84	82.9	80
Population mobile phone coverage, mobile RDD (%)[2-9,15]	94.1	48	68	95	84	94	75.5*	95	83	99.9	56.4	94	99	97	99
**Health system indicators**															
Current health expenditure per capita, PPP (current international $)[1]	2,198.88	75	208	1,187	712	1,204	1,111	2,310	211	3,521	212	3,998	5,087.38	10,921	2,652.95
Domestic general government health expenditure (% of current health expenditure)[1]	62.36	22.7	46	58.8	62.9	71.9	49.3	66.6	32.8	59.5	36.9	73.9	79.47	50.8	53.97
Out-of-pocket expenditure (% of current health expenditure)[1]	27.66	37.9	24.3	5.7	28.1	14.9	42.1	15.5	54.8	30.3	41.8	23.3	17.07	11.3	33.44
HAQ index (2019)[10]	59.9	31.2	33.4	44.6	60	61.1	52.5	64.7	39.2	86.3	33	89.6	83.3	80.6	83.9
**Health outcome indicators**															
Life expectancy at birth, total (years)[1]	76	65	63	65	74	75	70	78	70	83	68	82	81	77	80

Maternal mortality ratio (national estimate, per 100,000 live births)[1]	32	557 (2016)	377 (2014)	78 (2015)	102 (2012)	44 (2017)	36 (2016)	17 (2018)	143 (2017)	8 (2016)	217 (2015)	3 (2015)	8	19 (2016)	8 (2020)
Under-five mortality rate (per 1,000 live births)[1]	7	49	42	32	13	13	14	6	33	3	44	3	4	6	4
Population prevalence of hypertension in adults aged 30-79 years (crude)[11]	47.5	25.2	28.6	41.3	20.4	31.3	31.4	46.6	30.1	31	25.9	42.4	26.4	36.9	38.2
Age-adjusted prevalence of diabetes in adults aged 20-79 years[12]	5.4	5	4	10.8	4.8	8.3	16.9	9	9.6	6.8	6.2	6.4	6.3	10.7	6.4
COVID-19 cases (cumulative per million) (as of survey start date)[13]	213,323	3,995	6,256	67,194	107,026	119,498	56,673	282,041	31,531	585,732	27,726	421,886	362,791	289,885	511,380
COVID-19 deaths (cumulative per million) (a s of survey start date)[14]	2,855	61	105	1,707	6,273	2,703	2,597	2,154	374	650	100	3,104	3,287	3,175	3,560.05

*Population telephone coverage is greater than 80% when landlines are included.

Table references:

1. World Bank. (2023). *World Bank Open Data*. Available at: https://data.worldbank.org/ (Accessed: 26 April 2023).
2. Pew Research Center. (2016). *Smartphone ownership rates skyrocket in many emerging economies*. Pew Research Center's Global Attitudes Project. Available at: https://www.pewresearch.org/global/2016/02/22/smartphone-ownership-rates-skyrocket-in-many-emerging-economies-but-digital-divide-remains/ (Accessed: 26 April 2023).
3. Pew Research Center. (2019). *Non-mobile phone users in emerging countries: What hinders their access?* Pew Research Center: Internet, Science & Tech. Available at: https://www.pewresearch.org/internet/2019/11/20/non-mobile-phone-users-what-hinders-their-access/ (Accessed: 26 April 2023).
4. Pew Research Center. (2018). *Majorities in sub-Saharan Africa own mobile phones, but smartphone adoption is modest*. Pew Research Center's Global Attitudes Project. Available at: https://www.pewresearch.org/global/2018/10/09/majorities-in-sub-saharan-africa-own-mobile-phones-but-smartphone-adoption-is-modest/ (Accessed: 26 April 2023).
5. International Telecomunication Union (2023). *Core Households Indicators*. Available at: https://www.itu.int/en/ITU-D/Statistics/Documents/statistics/2019/CoreHouseholdIndicators.xlsx (Accessed: 26 April 2023).
6. Eurobarometer. (2021). *E-Communications in the Single Market.* Available at: https://europa.eu/eurobarometer/surveys/detail/2232 (Accessed: 26 April 2023).

7. Helgi Library. (2021). *Mobile Phone Penetration (As % of Population) in Laos*. Available at: https://www.helgilibrary.com/indicators/mobile-phone-penetration-as-of-population/laos/ (Accessed: 26 April 2023).
8. The Telegraph. (2023). *Mobile Phone Coverage in the UK Explained*. Available at: https://www.telegraph.co.uk/compare/mobile/mobile-phone-coverage (Accessed: 26 April 2023).
9. The Global Economy (2016). Argentina*: Mobile network coverage.* Available at: https://www.theglobaleconomy.com/Argentina/Mobile_network_coverage/ (Accessed: 26 April 2023).
10. GBD 2019 Healthcare Access and Quality Collaborators (2022). Assessing performance of the Healthcare Access and Quality Index, overall and by select age groups, for 204 countries and territories, 1990-2019: a systematic analysis from the Global Burden of Disease Study 2019. *The Lancet. Global health*, *10*(12).
11. World Health Organization. (2023). *The Global Health Observatory*. Available at: https://www.who.int/data/gho/data/indicators/indicator-details/GHO/prevalence-of-hypertension-among-adults-aged-30-79-years (Accessed: 26 April 2023).
12. International Diabetes Federation. (2023). *IDF Diabetes Atlas.* Available at: https://diabetesatlas.org/data/en/indicators/2/ (Accessed: 26 April 2023).
13. Our World in Data. (2022). Coronavirus (COVID-19) Cases - Statistics and Research. Our World in Data. Available at: https://ourworldindata.org/covid-cases (Accessed: 26 April 2023).
14. Our World in Data. (2022). Coronavirus (COVID-19) Deaths - Statistics and Research. Our World in Data. Available at: https://ourworldindata.org/covid-deaths (Accessed: 26 April 2023).
15. Pew Research Center. (2022). *Social media seen as mostly good for democracy across many nations, but US is a major outlier*. Pew Research Center: Global. Available at: https://www.pewresearch.org/global/2022/12/06/internet-smartphone-and-social-media-use-in-advanced-economies-2022/ (Accessed: 7 Sep 2023).
