# Supplementary material for: Population confidence in the health system in 15 countries: results from the first round of the People's Voice Survey
Source: Lancet Glob Health. 2023 Dec 11;12(1):e100–11. doi: 10.1016/S2214-109X(23)00499-0 (PMC10716625; doi:10.1016/S2214-109X(23)00499-0)
Supplement: Supplementary appendix 2 [file mmc2.pdf]

THE LANCET
Global Health

Supplementary appendix 2

This appendix formed part of the original submission and has been peer reviewed. We post it as supplied by the authors.

Supplement to: Kruk ME, Kapoor NR, Lewis TP, et al. Population confidence in the health system in 15 countries: results from the first round of the People's Voice Survey. *Lancet Glob Health* 2023; published online Dec 11. https://doi.org/10.1016/S2214-109X(23)00499-0.

Appendix 2: Additional Data

Appendix 1: Survey questions for health system quality and confidence measures

	Question	Response options	Dichotomization	
**Population reported health system quality**				
Quality of public primary care, key services	Thinking about the public or government-provided primary care system *[local system names]* in your opinion, how would you rate the quality of care provided for the following services?<br>A. Care for pregnant women, like antenatal care<br>B. Care for children, like well-child care and care for sick children<br>C. Care for ongoing or chronic conditions, like hypertension or diabetes<br>D. Care for mental health conditions, like depression or anxiety	For each service:<br>• Excellent<br>• Very Good<br>• Good<br>• Fair<br>• Poor	1	Excellent, Very good
			0	Good, Fair, Poor
Quality of national health system	Overall, how would you rate the quality of the government or public healthcare system in your country *[local system names]*?<br><br>Overall, how would you rate the quality of the private healthcare system in your country *[local system names]*?	For each system:<br>• Excellent<br>• Very Good<br>• Good<br>• Fair<br>• Poor	1	Excellent, Very good
			0	Good, Fair, Poor
**Confidence in the health system**				
Health security	How confident are you that you would…<br>A. Receive good quality healthcare if you became very sick?<br>B. Be able to afford the healthcare you needed if you became very sick?	For each item:<br>• Very confident<br>• Somewhat confident<br>• Not too confident<br>• Not at all confident	1	Very confident, Somewhat confident
			0	Not too confident, Not at all confident
Endorsement of health system	Thinking about the past two years, would you say your country's health system is getting better, staying the same, or getting worse?	• Getting better<br>• Staying the same<br>• Getting worse	1	Getting better
			0	Staying the same, Getting worse
	Which of these statements do you agree with the most?	• Our healthcare system has so much wrong with it that we need to completely rebuild it.<br>• There are some good things in our healthcare system, but major changes are needed to make it work better.<br>• On the whole, the system works pretty well and only minor changes are necessary to make it work better.	1	On the whole, the system works pretty well…
			0	There are some good things in our healthcare system…, Our healthcare system has so much wrong with it…
Government responsiveness to public input	How confident are you…<br>That the government considers the public's opinion when making decisions about the healthcare system?	• Very confident<br>• Somewhat confident<br>• Not too confident<br>• Not at all confident	1	Very confident, Somewhat confident
			0	Not too confident, Not at all confident
Government COVID-19 management	Overall, how would you rate the quality of the government or public healthcare system in your country?	• Excellent<br>• Very good<br>• Good<br>• Fair<br>• Poor	1	Excellent, Very good
			0	Good, Fair, Poor

In Korea, for quality ratings of primary care key services, the public system was not specified, and instead, respondents were asked about these services generally. For quality of national health system, further details on local system names are in appendix 2.

Appendix 2: Country specific response options for health system quality measures

Dimensions of health system endorsement	Ethiopia	Kenya	South Africa	Peru	Colombia	Mexico	Uruguay	Argentina	India	South Korea	Lao PDR	Greece	Italy	United Kingdom	United States
For the next set of questions, we are interested in your views on **[primary care services provided by public or government-provided primary care system]**.	Primary care services provided by the government	Government-provided primary care services, such as services from community health workers, clinics, health centers, and maternity and nursing homes	Primary care services provided by department of health facilities such as government hospitals and clinics	Care given in the centers, posts and health posts of the MINSA, not in the hospitals	Care given in the health centers of the Colombian public system, the district or municipal system	Public primary care services provided by the Mexican Institute for Social Security (IMSS) if usual source of care, or by Ministry of Health or State Health Services, otherwise	Care given in the health centers of the public system, not in hospitals or sanatoriums.	Care provided in the public health system's health center and "salitas," not in hospitals or sanatariums	Primary care services provided by the government	Healthcare provided by a government-run primary medical institution (e.g., a health center)	Government health services	Primary care services provided by public primary care system, for example the services provided by a public health center	Public primary care services provided by the National Health Service (SSN).	The National Health Service (NHS) or Health and Social care (HSC) (if Northern Ireland)	Public primary care services including those provided by Federally Qualified Health Centers (known as “free clinics”)
Overall, how would you rate the quality of the [**government or public healthcare system]** in your country?	Government or public healthcare system	Government or public healthcare system	Government or public healthcare system	MINSA public health system	Public health system	Secretaria de Salud o Servicios Estatales de Salud	Public health system	Public health system	Government or public healthcare system	Government or government-owned public health care system	Government or public hospitals and health centers	Government or public healthcare system	Services provided by the National Health Service (SSN)	The National Health Service (NHS) or Health and Social care (HSC) (if Northern Ireland)	Services provided by Federally Qualified Health Centers (known as “free clinics”)
Overall, how would you rate the quality of the **[private for-profit healthcare system]** in your country?	Private for-profit healthcare system	Private for-profit healthcare system	Private for-profit healthcare system	Private health system	Private health system	Private for-profit healthcare system	Private health system	Mendoza's OSEP	Private for-profit healthcare system	Privately owned for-profit healthcare system	Private for-profit hospitals and clinics	Private for-profit healthcare system	Private for-profit healthcare system	the private for-profit healthcare system	Private for-profit healthcare system
Overall, how would you rate the quality of the **[other]** in your country?	NGO or faith-based healthcare system	NGO or faith-based healthcare system	NGO or faith-based healthcare system	Social security health system (EsSalud)	NA	Mexican Institute for Social Security (IMSS), IMSS Bienestar (antes IMSS Prospera o IMSS Opportunidades)	Health system of mutuals or social works	Other "obras sociales"	NGO or faith-based healthcare system	Private healthcare run by non-profit organizations or religious organizations	NA	NGO or faith-based healthcare system	NA	NA	NA

Appendix 3: Country specific income categorization

Country	Income categories	Income values
**Argentina (Mendoza)**	Lowest income group	No income - 59,999 pesos
	Middle income group	60,000 - 129,999 pesos
	Highest income group	130,000 pesos or more
**Colombia**	Lowest income group	No income - 200,000 pesos
	Middle income group	200,000 - 800,000 pesos
	Highest income group	800,000 pesos or more
**Ethiopia**	Lowest income group	Less than 1,000 Eth.Birr
	Middle income group	1,000 - 3,000 Eth.Birr
	Highest income group	3,001 Eth.Birr or more
**Greece**	Lowest income group	Less than 270 euros
	Middle income group	271 - 3,300 euros
	Highest income group	More than 3,301 euros
**India**	Lowest income group	Less than 3,000 - 10,000 INR
	Middle income group	10,001 - 30,000 INR
	Highest income group	30,001 INR or more
**Italy**	Lowest income group	Less than 10,000 - 15,000 euros
	Middle income group	15,000 - 26,000 euros
	Highest income group	26,000 euros or more
**Kenya**	Lowest income group	Less than Ksh 15,572
	Highest income group	Ksh 15,573 or more
**Lao PDR**	Lowest income group	Less than 1,000,000 - 1,500,000
	Middle income group	1,500,001 to 2,500,000
	Highest income group	2,500,001 or more
**Mexico**	Lowest income group	Less than 6,500 pesos
	Middle income group	6,500 - 10,000 pesos
	Highest income group	10,000 pesos or more
**Peru**	Lowest income group	No income - S/. 1,000
	Middle income group	S/. 1,000 - 2,500
	Highest income group	S/. 2,500 or more
**Republic of Korea**	Lowest income group	Less than 500,000 - 3,000,000 won
	Middle income group	3,000,000 - 5,000,000 won
	Highest income group	5,000,000 won or more
**South Africa**	Lowest income group	No income - 1,500 rand
	Middle income group	1,501 - 6,000 rand
	Highest income group	6,001 rand or more
**United States**	Lowest income group	Less than $26,000 - $36,000
	Middle income group	$36,000 - $65,000
	Highest income group	$65,000 or more
**Uruguay**	Lowest income group	No income - 30,000 pesos
	Middle income group	30,000 - 50,000 pesos
	Highest income group	50,000 pesos or more
**United Kingdom**	Lowest income group	Less than £14,000 - £25,000
	Middle income group	£25,000 - £42,000
	Highest income group	£42,000 or more

In Kenya, there was no middle income group. Lao PDR is Lao People's Democratic Republic.

Appendix 4: PVS Summary Statistics

	ET (N = 2779)	KE (N = 2305)	ZA (N = 2036)	PE (N = 1255)	CO (N = 1237)	ME (N = 1002)	UY (N = 1237)	AR (N = 1190)	LA (N = 2007)	IN (N = 2004)	KR (N = 2000)	GR (N = 2015)	IT (N = 1001)	GB (N = 1677)	US (N = 1500)	Total (N = 25245)
**<u>Demographics and health</u>**																
**Gender**																
Female	1388 (49.9%)	1164 (50.5%)	1041 (51.2%)	627 (50.0%)	641 (51.9%)	526 (52.7%)	644 (52.2%)	729 (61.3%)	1033 (51.5%)	961 (48.0%)	1007 (50.4%)	1033 (51.4%)	520 (52.0%)	866 (51.7%)	763 (51.2%)	12937 (51.3%)
**Age**																
Median (Q1, Q3)	32.0 (23.5, 45.0)	32.0 (24.0, 44.0)	37.0 (28.0, 49.0)	40.0 (29.0, 54.0)	40.0 (28.0, 55.0)	40.0 (28.0, 55.0)	45.0 (32.0, 60.0)	47.0 (36.0, 61.0)	38.0 (27.0, 50.0)	36.0 (26.0, 50.0)	49.0 (35.0, 61.0)	47.0 (34.0, 64.0)	53.0 (38.0, 65.0)	48.0 (33.0, 63.0)	46.0 (31.0, 63.0)	40.0 (28.0, 55.0)
**Highest level of education completed**																
None	1378 (49.6%)	339 (14.7%)	105 (5.1%)	70 (5.5%)	109 (8.8%)	38 (3.7%)	25 (2.0%)	29 (2.4%)	133 (6.6%)	203 (10.2%)	0 (0.0%)	33 (1.6%)	0 (0.0%)	9 (0.5%)	3 (0.2%)	2466 (9.8%)
Primary	851 (30.6%)	1154 (50.2%)	1029 (50.6%)	408 (32.6%)	503 (40.6%)	258 (25.7%)	523 (42.3%)	298 (25.0%)	950 (47.3%)	496 (25.0%)	4 (0.2%)	621 (30.9%)	184 (18.3%)	6 (0.3%)	72 (4.7%)	7350 (29.2%)
Secondary	333 (12.0%)	563 (24.4%)	670 (32.9%)	563 (44.9%)	359 (29.0%)	532 (53.0%)	537 (43.4%)	468 (39.4%)	622 (31.0%)	542 (27.3%)	583 (29.1%)	836 (41.6%)	662 (66.2%)	393 (23.6%)	502 (33.4%)	8157 (32.4%)
Post-secondary	220 (7.9%)	246 (10.7%)	232 (11.4%)	214 (17.0%)	267 (21.5%)	176 (17.5%)	153 (12.3%)	395 (33.2%)	305 (15.2%)	744 (37.5%)	1415 (70.7%)	521 (25.9%)	155 (15.5%)	1253 (75.5%)	925 (61.6%)	7211 (28.6%)
**Place of residence**																
Urban	825 (29.7%)	758 (32.8%)	1398 (68.8%)	1033 (82.3%)	1059 (85.9%)	773 (78.0%)	1138 (92.5%)	1116 (93.9%)	678 (33.7%)	980 (49.4%)	1737 (86.8%)	1519 (75.8%)	953 (95.2%)	1516 (90.4%)	1309 (87.3%)	16783 (66.7%)
**Income**																
Lowest income	1069 (41.9%)	1366 (69.3%)	764 (38.6%)	637 (51.5%)	386 (31.6%)	537 (58.2%)	545 (44.9%)	486 (43.2%)	507 (28.4%)	750 (47.7%)	617 (30.8%)	57 (3.8%)	415 (45.6%)	496 (32.1%)	503 (33.5%)	9126 (39.7%)
Middle income	680 (26.6%)	-	699 (35.3%)	409 (33.1%)	421 (34.5%)	193 (20.8%)	322 (26.5%)	387 (34.4%)	395 (22.1%)	599 (38.1%)	721 (36.0%)	1387 (92.3%)	268 (29.4%)	469 (30.4%)	380 (25.3%)	7322 (31.8%)
Highest income	804 (31.5%)	605 (30.7%)	516 (26.1%)	191 (15.4%)	413 (33.8%)	195 (21.0%)	347 (28.6%)	252 (22.4%)	882 (49.5%)	223 (14.2%)	664 (33.2%)	60 (4.0%)	227 (24.9%)	580 (37.6%)	616 (41.1%)	6567 (28.5%)
**Self-rated health**																
Very good/Excellent	1103 (39.7%)	850 (36.9%)	779 (38.3%)	154 (12.2%)	288 (23.3%)	223 (22.4%)	346 (28.0%)	401 (33.7%)	268 (13.3%)	484 (24.1%)	491 (24.5%)	815 (40.6%)	320 (31.9%)	692 (41.4%)	683 (45.5%)	7889 (31.3%)
**Has chronic illness**																
Yes	365 (13.1%)	362 (15.7%)	555 (27.2%)	313 (24.9%)	335 (27.1%)	235 (23.4%)	535 (43.2%)	492 (41.4%)	480 (23.9%)	292 (14.6%)	786 (39.2%)	661 (32.9%)	322 (32.2%)	841 (50.9%)	611 (40.7%)	7177 (28.5%)
**Unmet healthcare need**																
Yes	305 (11.0%)	492 (21.4%)	194 (9.5%)	323 (25.8%)	247 (19.9%)	68 (6.7%)	150 (12.1%)	234 (19.7%)	334 (16.6%)	123 (6.1%)	120 (5.9%)	164 (8.1%)	62 (6.2%)	369 (22.4%)	282 (18.8%)	3460 (13.7%)
**<u>Quality of health system</u>**																
**Quality of usual source of care**																
Very good/Excellent	590 (36.8%)	618 (43.2%)	720 (56.4%)	248 (29.1%)	318 (34.5%)	337 (44.5%)	567 (53.7%)	589 (61.0%)	257 (16.7%)	260 (29.0%)	544 (43.8%)	629 (63.7%)	301 (45.5%)	679 (50.5%)	866 (71.5%)	7517 (44.9%)
**Quality of last visit**																
Very good/Excellent	729 (43.3%)	762 (42.5%)	831 (54.4%)	319 (33.4%)	374 (36.2%)	392 (50.0%)	650 (60.0%)	677 (65.9%)	357 (27.5%)	426 (39.6%)	557 (29.4%)	1248 (74.0%)	484 (60.8%)	970 (67.5%)	1019 (74.0%)	9789 (50.4%)

**Quality of public primary care: women**																
Very good/Excellent	1265 (46.8%)	790 (37.9%)	626 (33.7%)	203 (16.5%)	296 (25.9%)	259 (26.9%)	377 (34.4%)	339 (37.1%)	490 (25.5%)	634 (36.5%)	485 (24.2%)	307 (22.2%)	300 (35.1%)	496 (47.5%)	485 (45.6%)	7345 (33.4%)
**Quality of public primary care: children**																
Very good/Excellent	1048 (38.9%)	862 (38.7%)	706 (36.2%)	206 (16.7%)	315 (26.5%)	271 (28.0%)	415 (36.6%)	389 (38.8%)	504 (26.0%)	586 (33.5%)	656 (32.8%)	388 (27.4%)	336 (38.0%)	570 (49.8%)	532 (47.1%)	7775 (34.3%)
**Quality of public primary care: chronic conditions**																
Very good/Excellent	602 (23.9%)	580 (28.2%)	685 (35.1%)	119 (9.6%)	245 (20.6%)	206 (21.2%)	311 (27.5%)	305 (31.7%)	214 (11.8%)	458 (27.6%)	693 (34.6%)	309 (21.9%)	249 (27.9%)	467 (37.7%)	465 (40.4%)	5904 (26.6%)
**Quality of public primary care: mental health**																
Very good/Excellent	464 (19.8%)	487 (26.3%)	562 (30.1%)	125 (10.1%)	194 (16.9%)	140 (15.2%)	175 (15.9%)	170 (19.6%)	179 (10.9%)	428 (27.3%)	435 (21.7%)	153 (12.3%)	107 (14.3%)	231 (18.2%)	280 (25.2%)	4122 (19.7%)
**Quality of national health system, public**																
Very good/Excellent	969 (34.9%)	593 (25.8%)	573 (28.1%)	187 (14.9%)	186 (15.0%)	185 (18.9%)	334 (27.3%)	348 (29.7%)	514 (25.6%)	420 (21.9%)	848 (42.4%)	168 (8.6%)	207 (20.9%)	704 (42.3%)	284 (18.9%)	6511 (26.1%)
**Quality of national health system: private**																
Very good/Excellent	892 (33.1%)	1336 (59.0%)	1089 (54.4%)	230 (18.5%)	268 (22.5%)	254 (25.7%)	427 (35.7%)	309 (30.2%)	509 (26.1%)	528 (27.6%)	652 (32.5%)	572 (31.3%)	319 (33.7%)	672 (54.3%)	616 (41.1%)	8664 (36.2%)
**Quality of social security system**																
Very good/Excellent	-	-	-	194 (15.6%)	-	-	-	-	-	-	-	-	-	-	-	194 (15.6%)
**Quality of mutual healthcare system**																
Very good/Excellent	-	-	-	-	-	-	354 (29.3%)	-	-	-	-	-	-	-	-	354 (29.3%)
**Quality of IMSS**																
Very good/Excellent	-	-	-	-	-	200 (20.3%)	-	-	-	-	-	-	-	-	-	200 (20.3%)
**Quality of IMSS Bienestar**																
Very good/Excellent	-	-	-	-	-	200 (21.0%)	-	-	-	-	-	-	-	-	-	200 (21.0%)
**Quality of OSEP Mendoza**																
Very good/Excellent	-	-	-	-	-	-	-	103 (11.0%)	-	-	-	-	-	-	-	103 (11.0%)

**Quality of ‘obras sociales’**																
Very good/Excellent	-	-	-	-	-	-	-	114 (21.0%)	-	-	-	-	-	-	-	114 (21.0%)
**Quality of PAMI**																
Very good/Excellent	-	-	-	-	-	-	-	75 (12.6%)	-	-	-	-	-	-	-	75 (12.6%)
**<u>Confidence in health system</u>**																
**Health security: get good quality care when needed**																
Somewhat confident/Very confident	2197 (79.5%)	1846 (80.5%)	1363 (67.0%)	473 (37.7%)	549 (44.9%)	822 (82.6%)	793 (65.1%)	765 (64.8%)	1663 (83.0%)	1622 (84.1%)	1569 (78.4%)	831 (42.9%)	702 (70.9%)	1228 (74.2%)	1234 (82.4%)	17648 (70.7%)
**Health security: afford care when needed**																
Somewhat confident/Very confident	1520 (54.8%)	1093 (47.6%)	1164 (57.3%)	619 (49.6%)	529 (43.3%)	734 (74.0%)	520 (43.3%)	492 (42.1%)	1641 (82.1%)	1453 (76.4%)	1307 (65.3%)	512 (26.0%)	699 (70.4%)	916 (56.5%)	925 (61.9%)	14117 (56.7%)
**Health security: get and afford care when needed**																
Somewhat confident/Very confident	1333 (48.3%)	984 (43.0%)	986 (48.5%)	329 (26.4%)	372 (30.7%)	650 (65.8%)	440 (37.1%)	382 (32.9%)	1425 (71.3%)	1293 (69.2%)	1188 (59.4%)	401 (21.0%)	629 (63.9%)	786 (48.8%)	862 (57.7%)	12051 (48.8%)
**Endorsement: health system getting better in past two years**																
Getting worse	243 (8.8%)	530 (23.2%)	687 (34.1%)	458 (36.8%)	408 (33.2%)	219 (21.9%)	303 (24.9%)	457 (39.8%)	29 (1.4%)	191 (9.9%)	148 (7.3%)	782 (40.1%)	516 (52.3%)	1201 (71.6%)	533 (35.5%)	6699 (26.9%)
Staying the same	376 (13.7%)	432 (18.9%)	493 (24.5%)	464 (37.2%)	404 (32.8%)	394 (39.4%)	406 (33.3%)	351 (30.6%)	265 (13.3%)	438 (22.8%)	866 (43.2%)	726 (37.2%)	341 (34.5%)	379 (22.6%)	742 (49.4%)	7067 (28.4%)
Getting better	2129 (77.5%)	1325 (57.9%)	835 (41.4%)	325 (26.0%)	417 (33.9%)	386 (38.7%)	508 (41.7%)	341 (29.7%)	1694 (85.3%)	1289 (67.3%)	989 (49.4%)	444 (22.7%)	130 (13.1%)	98 (5.8%)	226 (15.1%)	11129 (44.7%)
**Endorsement: health system works well, only minor changes needed**																
Minor changes	829 (30.2%)	582 (25.4%)	434 (21.3%)	181 (14.4%)	230 (18.6%)	227 (22.9%)	321 (26.0%)	199 (16.9%)	744 (37.7%)	622 (33.6%)	822 (41.1%)	220 (11.1%)	311 (31.1%)	240 (14.5%)	345 (23.0%)	6300 (25.3%)
**<u>Political and social context</u>**																
**Confidence that the government considers public's opinion**																

Somewhat confident/Very confident	2181 (79.6%)	1410 (62.5%)	1047 (51.6%)	495 (39.5%)	476 (38.7%)	731 (73.7%)	440 (37.0%)	317 (27.2%)	1553 (79.0%)	1441 (76.5%)	1059 (52.9%)	501 (26.3%)	396 (40.8%)	427 (25.9%)	536 (35.7%)	13001 (52.6%)
**Government's management of the COVID-19 pandemic**																
Very good/Excellent	1488 (53.9%)	1130 (49.6%)	792 (39.4%)	163 (13.0%)	299 (24.2%)	255 (25.6%)	675 (54.9%)	460 (39.0%)	905 (45.8%)	733 (37.2%)	616 (30.8%)	448 (22.9%)	248 (25.0%)	382 (23.0%)	322 (21.5%)	8910 (35.7%)

Appendix 5: Association between quality of and confidence in health system and sociodemographic factors by country (values for Exhibit 3)

	Ethiopia		Kenya		South Africa		Peru		Colombia		Mexico		Uruguay		Argentina		Lao PDR		India		Rep of Korea		Greece		Italy		United Kingdom		United States	
**Quality of Public System**																														
High income	0.8	[0.7,1.0]	1.2	[0.9,1.4]	0.6	[0.5,0.8]	0.5	[0.2,1.0]	0.4	[0.3,0.7]	1.0	[0.6,1.5]	0.6	[0.4,0.8]	0.7	[0.5,0.9]	1.1	[0.9,1.4]	0.8	[0.5,1.1]	1.2	[0.9,1.4]	1.6	[0.9,2.8]	1.2	[0.8,1.7]	0.8	[0.7,1.0]	0.5	[0.4,0.7]
High education	0.6	[0.5,0.8]	0.5	[0.4,0.6]	0.5	[0.4,0.7]	0.2	[0.1,0.4]	0.4	[0.2,0.6]	0.8	[0.5,1.2]	1.0	[0.7,1.4]	0.9	[0.7,1.2]	0.8	[0.6,1.0]	0.8	[0.6,1.0]	0.9	[0.8,1.2]	0.8	[0.5,1.2]	1.1	[0.8,1.7]	0.9	[0.7,1.1]	0.7	[0.5,1.0]
Urban	0.7	[0.6,1.0]	0.9	[0.8,1.2]	0.7	[0.6,0.9]	1.2	[0.6,2.5]	0.9	[0.6,1.6]	1.0	[0.6,1.6]	1.0	[0.6,1.7]	1.6	[0.8,3.0]	0.8	[0.6,1.0]	0.7	[0.6,0.9]	0.8	[0.6,1.1]	0.8	[0.5,1.3]	0.8	[0.4,1.9]	1.0	[0.7,1.3]	1.2	[0.8,1.8]
Under 30 years	1.0	[0.8,1.2]	1.3	[1.0,1.6]	1.0	[0.8,1.2]	0.9	[0.6,1.4]	0.7	[0.5,1.1]	1.1	[0.7,1.6]	0.7	[0.5,1.1]	0.8	[0.5,1.2]	0.9	[0.7,1.2]	1.2	[0.9,1.5]	1.1	[0.8,1.4]	0.7	[0.4,1.4]	1.8	[1.1,2.9]	0.8	[0.6,1.0]	1.0	[0.7,1.3]
Female	1.3	[1.1,1.5]	1.2	[1.0,1.5]	0.9	[0.8,1.2]	0.8	[0.5,1.2]	0.9	[0.6,1.3]	0.6	[0.4,0.9]	0.7	[0.6,1.0]	0.9	[0.7,1.2]	1.0	[0.8,1.2]	0.7	[0.5,0.9]	0.8	[0.6,0.9]	1.0	[0.7,1.5]	0.7	[0.5,0.9]	0.9	[0.7,1.0]	1.0	[0.7,1.3]
N	2602		2076		1948		1234		1206		900		1183		1102		1819		1589		1998		1612		892		1542		1485	
**Confidence can get and afford care**																														
High income	1.0	[0.9,1.2]	1.6	[1.3,1.9]	1.3	[1.0,1.5]	1.2	[0.9,1.7]	0.7	[0.5,1.0]	1.1	[0.8,1.5]	1.6	[1.2,2.1]	2.3	[1.6,3.1]	1.3	[1.0,1.6]	1.2	[0.9,1.8]	1.6	[1.3,2.0]	2.6	[1.8,3.9]	1.7	[1.2,2.3]	2.0	[1.6,2.5]	1.7	[1.4,2.2]
High education	0.8	[0.7,1.0]	1.0	[0.8,1.2]	0.8	[0.7,1.0]	0.6	[0.5,0.8]	0.7	[0.5,1.0]	0.8	[0.6,1.1]	1.1	[0.8,1.5]	1.0	[0.7,1.3]	1.1	[0.9,1.4]	1.0	[0.8,1.3]	1.1	[0.9,1.3]	1.2	[0.9,1.6]	1.0	[0.8,1.4]	1.2	[0.9,1.6]	0.8	[0.6,1.0]
Urban	0.8	[0.7,1.0]	0.9	[0.8,1.1]	0.9	[0.7,1.1]	1.0	[0.7,1.6]	1.1	[0.8,1.7]	1.2	[0.9,1.8]	0.9	[0.6,1.5]	0.9	[0.5,1.6]	0.8	[0.7,1.0]	1.0	[0.8,1.2]	0.9	[0.7,1.2]	1.4	[1.0,2.2]	0.7	[0.3,1.4]	0.9	[0.7,1.3]	1.2	[0.8,1.6]
Under 30 years	1.1	[0.9,1.3]	1.0	[0.9,1.2]	0.8	[0.7,1.0]	1.2	[0.9,1.7]	1.1	[0.8,1.4]	1.0	[0.8,1.4]	1.9	[1.4,2.7]	1.2	[0.8,1.9]	0.7	[0.5,0.9]	1.1	[0.8,1.3]	1.0	[0.8,1.3]	0.8	[0.6,1.3]	1.0	[0.6,1.6]	1.3	[0.9,1.7]	0.5	[0.4,0.7]
Female	0.9	[0.8,1.1]	1.2	[1.0,1.4]	0.9	[0.7,1.1]	0.9	[0.7,1.1]	0.8	[0.6,1.0]	0.9	[0.7,1.2]	0.8	[0.6,1.0]	0.7	[0.5,0.9]	0.9	[0.7,1.1]	1.3	[1.0,1.6]	0.8	[0.7,1.0]	0.7	[0.5,0.9]	0.7	[0.5,0.9]	0.8	[0.7,1.0]	0.8	[0.6,1.0]
N	2595		2075		1945		1231		1196		900		1161		1091		1809		1564		1999		1585		886		1493		1482	
**Health system getting better in past two years**																														
High income	0.8	[0.6,1.0]	1.0	[0.8,1.2]	0.6	[0.5,0.7]	0.9	[0.7,1.3]	0.9	[0.7,1.2]	0.9	[0.6,1.2]	0.6	[0.4,0.8]	0.8	[0.6,1.1]	1.0	[0.7,1.3]	0.8	[0.6,1.2]	1.2	[1.0,1.4]	1.3	[0.8,2.0]	0.7	[0.4,1.2]	0.8	[0.5,1.4]	0.6	[0.4,0.8]
High education	0.9	[0.7,1.1]	1.1	[0.9,1.3]	0.7	[0.5,0.8]	0.7	[0.5,1.0]	0.6	[0.5,0.9]	0.9	[0.6,1.2]	0.7	[0.5,1.0]	0.6	[0.5,0.9]	0.7	[0.5,0.9]	1.4	[1.1,1.7]	0.9	[0.8,1.1]	0.8	[0.6,1.1]	1.3	[0.8,2.0]	0.9	[0.5,1.5]	0.9	[0.6,1.2]
Urban	0.8	[0.7,1.1]	1.5	[1.2,1.8]	0.8	[0.6,1.0]	0.8	[0.5,1.2]	1.3	[0.9,2.0]	1.1	[0.7,1.5]	0.9	[0.6,1.5]	1.4	[0.8,2.5]	1.3	[1.0,1.7]	1.3	[1.1,1.7]	1.2	[0.9,1.5]	1.3	[0.9,1.8]	1.3	[0.4,3.7]	1.4	[0.6,3.6]	2.8	[1.5,5.1]
Under 30 years	1.1	[0.9,1.4]	1.5	[1.2,1.8]	1.2	[1.0,1.4]	1.3	[1.0,1.7]	0.9	[0.7,1.2]	0.9	[0.6,1.2]	1.1	[0.8,1.5]	1.2	[0.8,2.0]	0.6	[0.4,0.8]	1.0	[0.8,1.2]	0.7	[0.5,0.9]	0.9	[0.6,1.3]	1.2	[0.6,2.2]	1.8	[1.0,3.3]	0.8	[0.5,1.1]
Female	1.4	[1.2,1.7]	0.9	[0.7,1.0]	0.9	[0.7,1.1]	1.0	[0.8,1.3]	0.9	[0.7,1.1]	0.6	[0.4,0.8]	0.7	[0.5,0.9]	1.0	[0.7,1.3]	0.9	[0.7,1.1]	1.0	[0.8,1.2]	1.0	[0.8,1.2]	0.7	[0.6,0.9]	0.7	[0.5,1.1]	0.8	[0.5,1.3]	0.6	[0.5,0.9]

N	2587		2074		1937		1231		1205		912		1182		1081		1815		1591		1999		1590		886		1546		1486	
**Health system works well, only minor changes needed**																														
High income	0.9	[0.7,1.1]	0.9	[0.7,1.1]	0.7	[0.5,0.9]	0.4	[0.2,0.7]	0.8	[0.5,1.1]	0.6	[0.4,1.0]	0.9	[0.7,1.2]	0.7	[0.5,1.1]	0.9	[0.7,1.1]	1.0	[0.7,1.4]	1.3	[1.0,1.5]	1.3	[0.7,2.3]	1.2	[0.9,1.7]	0.7	[0.5,1.0]	1.4	[1.0,1.9]
High education	0.4	[0.3,0.5]	0.5	[0.4,0.7]	0.7	[0.6,1.0]	0.3	[0.2,0.6]	0.4	[0.2,0.6]	0.5	[0.3,0.7]	0.9	[0.7,1.3]	0.6	[0.4,0.9]	0.7	[0.6,0.9]	0.9	[0.7,1.2]	1.0	[0.8,1.2]	0.6	[0.5,0.9]	1.1	[0.8,1.5]	0.9	[0.6,1.2]	0.5	[0.4,0.7]
Urban	1.0	[0.8,1.3]	0.7	[0.6,0.9]	0.7	[0.6,0.9]	0.6	[0.4,1.0]	0.8	[0.5,1.2]	1.1	[0.7,1.8]	0.5	[0.3,0.8]	1.2	[0.6,2.4]	1.1	[0.9,1.3]	1.0	[0.8,1.3]	1.1	[0.8,1.4]	0.7	[0.5,1.1]	0.6	[0.3,1.1]	1.0	[0.6,1.7]	0.8	[0.6,1.2]
Under 30 years	1.4	[1.1,1.7]	1.3	[1.0,1.6]	1.2	[0.9,1.5]	0.8	[0.5,1.2]	0.6	[0.4,0.9]	1.2	[0.8,1.8]	0.8	[0.6,1.3]	0.9	[0.5,1.5]	0.9	[0.7,1.1]	0.7	[0.6,0.9]	0.6	[0.5,0.8]	0.9	[0.5,1.5]	1.2	[0.7,1.8]	0.9	[0.6,1.4]	0.3	[0.2,0.4]
Female	1.1	[0.9,1.4]	0.9	[0.8,1.2]	1.0	[0.8,1.2]	1.0	[0.7,1.5]	1.1	[0.8,1.5]	0.8	[0.6,1.1]	0.7	[0.5,0.9]	0.8	[0.6,1.1]	0.8	[0.7,1.0]	1.0	[0.8,1.3]	1.2	[1.0,1.4]	0.7	[0.5,0.9]	0.8	[0.6,1.0]	0.7	[0.5,0.9]	0.6	[0.5,0.8]
N	2598		2078		1948		1235		1208		909		1196		1111		1811		1564		1998		1609		899		1537		1485	

Exponentiated coefficients; 95% confidence intervals in brackets

Appendix 6: Association between quality of private system and sociodemographic factors in Republic of Korea and United States

	Rep of Korea		United States	
**Quality of private system**				
	**OR**	**CI**	**OR**	**CI**
High income	1.4	[1.1,1.7]	1.0	[0.8,1.3]
High education	1.0	[0.8,1.3]	1.1	[0.9,1.4]
Urban	0.8	[0.6,1.0]	1.1	[0.8,1.6]
Under 30 years	1.1	[0.8,1.4]	0.6	[0.5,0.8]
Female	0.7	[0.6,0.9]	0.7	[0.6,0.9]
N	1999		1484	

Exponentiated coefficients; 95% confidence intervals in brackets
